# Topoisomerase III limits RecA-dependent DNA amplification in the chromosome terminus with RecG

**DOI:** 10.1093/nar/gkag572

**Published:** 2026-06-08

**Authors:** Ali Dadras, Armelle Le Campion, Marc Drolet

**Affiliations:** Département de microbiologie, infectiologie et immunologie, Faculté de médecine, Université de Montréal, Montréal, P. Québec H3C 3J7, Canada; Institut Courtois d’innovation biomédicale, Faculté de médecine, Université de Montréal, Montréal, P. Québec H3C 3J7, Canada; Département de microbiologie, infectiologie et immunologie, Faculté de médecine, Université de Montréal, Montréal, P. Québec H3C 3J7, Canada; Institut Courtois d’innovation biomédicale, Faculté de médecine, Université de Montréal, Montréal, P. Québec H3C 3J7, Canada

## Abstract

Topoisomerase (topo) III is a ubiquitous type IA enzyme whose role in suppressing inappropriate recombination is well established in eukaryotes but remains poorly defined in prokaryotes. In *Escherichia coli*, deletion of *topB* (encoding topo III) in *topA* mutants (topo I, which normally prevents R-loop formation) leads to pronounced *Ter*/Tus barrier-dependent DNA amplification in the chromosomal terminus region. In *recG* mutants, this phenotype was recently attributed to RecA-mediated D-loop formation at DNA double-strand ends (DSEs) generated at *Ter*/Tus barriers, triggering replication restart in the incorrect orientation (re-replication). Here, our data indicate that, as in *recG* mutants, RecA drives repeated cycles of *Ter*/Tus barrier-dependent DSE formation and re-replication, thereby promoting chromosomal terminus DNA amplification in *topA topB* cells. Deletion of *topB* enhances *Ter*/Tus-dependent amplification in *rnhA* cells but not in *recG* cells. Moreover, deletion of *recG* or *topB*, but not *recQ*, markedly exacerbates the replication-completion defect of *topA* mutants, unless *recA* is deleted or topo IV is overproduced. Together, these findings suggest that topo III and RecG cooperate to restrain RecA-mediated re-replication at sites of DSE formation, thereby preventing pathological DNA amplification.

## Graphical Abstract

**Graphical Abstract:**
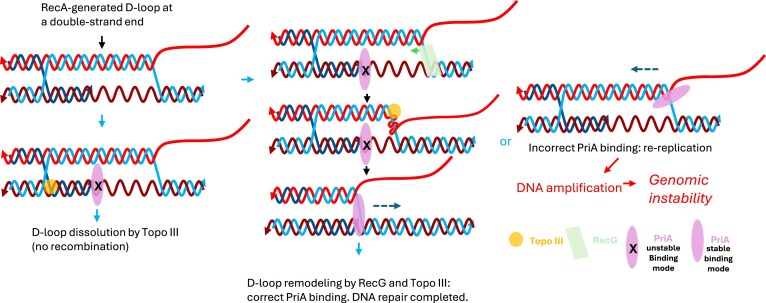

## Introduction

Topological issues such as underwinding, overwinding (supercoiling), and strand entanglement are inherent to the DNA double helix and must be resolved during transcription, replication, and repair [1]. DNA topoisomerases achieve this by cutting one DNA strand (type I) or both strands (type II) and relaxing topological stress through strand passage (type IA, IIA/B) or controlled rotation (type IB) [1, 2]. These enzymes are therefore essential for genome function and stability.

Type IA topoisomerases are the only universally conserved topoisomerases [3]. They act on single-stranded DNA to relax and decatenate DNA. Within this family, topo I is present in all bacteria but absent from archaea and eukaryotes, whereas topo III is found in most bacteria and in all archaea and eukaryotes. The canonical representatives are the *Escherichia coli* enzymes topo I (*topA*) and topo III (*topB*). Topo III primarily functions as a decatenase [4], whereas topo I mainly relaxes negative supercoils [5]. Single-molecule studies show that topo I possesses a rapidly cycling DNA gate that promotes efficient relaxation of negative supercoiling, while the slower gate-closing of topo III favors DNA capture and thus effective decatenation [6]. *In vivo*, a major role of topo I is to relax transcription-induced negative supercoiling [7] immediately behind the elongating RNA polymerase (RNAP) [8], thereby preventing R-loop formation [9, 10].

*Escherichia coli* topo III contains a distinctive structural element known as the “decatenation loop”, which is crucial for its strong decatenation activity [11]. In *E. coli*, its only well-established role is the removal of precatenanes that form behind replication forks [12, 13], although topo IV functions as the primary replicative decatenase [14]. Human cells encode two topo III enzymes, topo IIIα and topo IIIβ. Unlike *E. coli* topo III, human topo IIIα has a clearly defined role in suppressing inappropriate homologous recombination [15, 16]. This activity depends on its interaction with the OB-fold protein Rmi1, which serves as the functional equivalent of the decatenation loop in bacterial topo III and is required for topo IIIα’s decatenation function [17]. Topo IIIα participates in several recombination processes, including the dissolution of double-Holliday junctions (dHJs)—a reaction that also requires the helicase BLM, the evolutionary homolog of *E. coli* RecQ—as well as the dissolution of D-loops and related intermediates [15, 16]. Although BLM physically associates with topo IIIα, an analogous interaction between RecQ and *E. coli* topo III has not been observed, even though RecQ can modulate topo III activity *in vitro* [18, 19]. Genetic evidence suggesting a functional RecQ–topo III interaction in recombination in *E. coli* has been reported [20]; however, subsequent studies failed to substantiate this interaction [21, 22]. Moreover, biochemical analyses show that BLM more closely resembles the *E. coli* helicase RecG than RecQ, and RecG can even suppress genomic instability phenotypes in human cells lacking BLM [23].

Major homologous recombination hotspots have been identified in the *E. coli* genome through nucleotide-level mapping of double-strand ends (DSEs) and Holliday junctions (HJs), all of which cluster in the chromosomal terminus (Ter) region [24]. These sites, termed “fragile sites,” parallel human chromosomal regions that appear as gaps or breaks after replication stress and are known contributors to genome instability in cancer and developmental disorders [25]. In *E. coli*, fragile-site DNA breaks arise through two main mechanisms: shearing of unsegregated sister chromosomes during cell division, producing double-strand breaks (DSBs), and replication fork collapse at *Ter*/Tus barriers, generating one-ended breaks (DSEs) [24].

**Figure 1.**
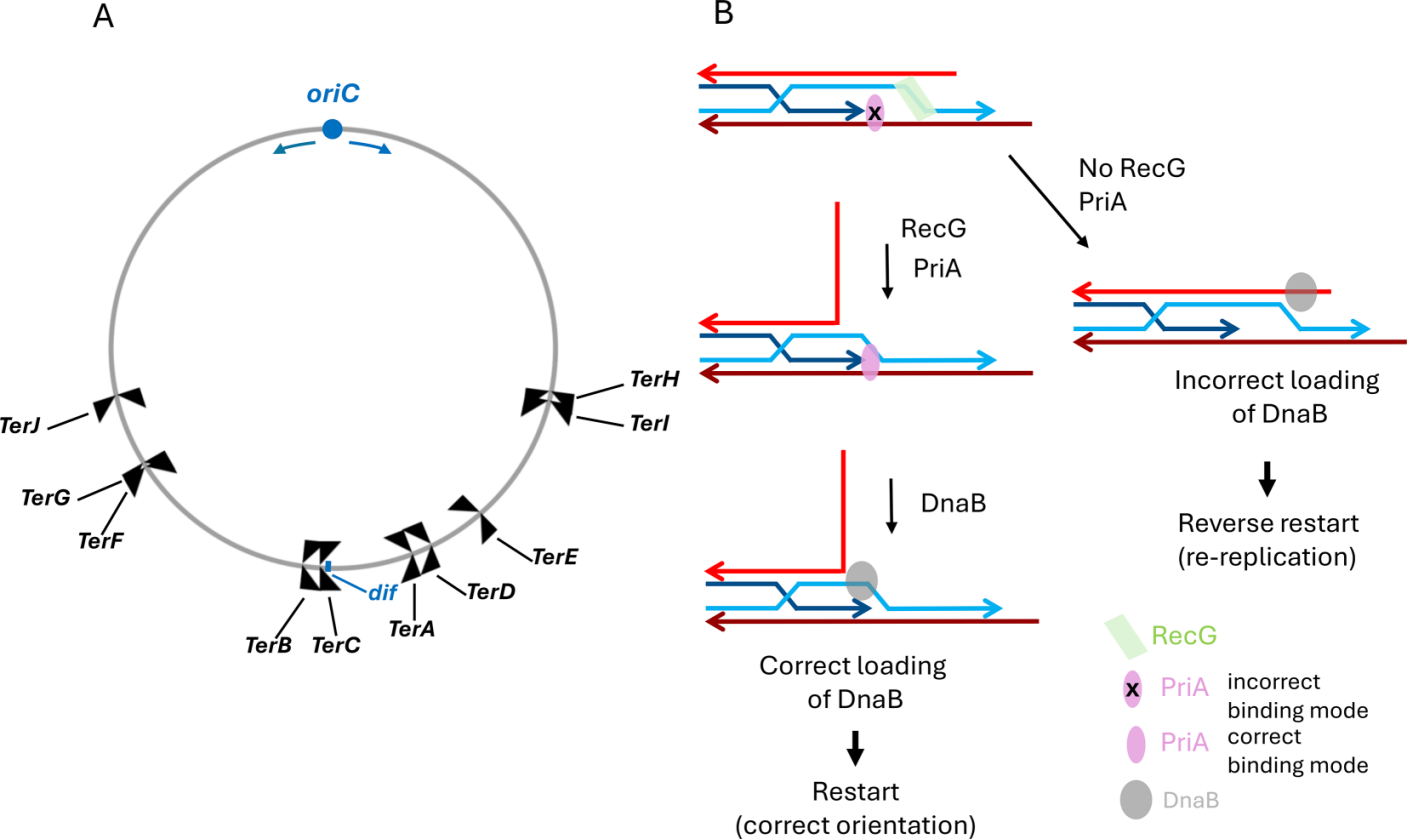
The chromosome terminus region (Ter) and re-replication. (**A**) Schematic representation of the circular *E. coli* chromosome. Replication initiates at *oriC* (blue dot) proceeds bidirectionally (blue arrows). The two replication forks converge in the terminus region, opposite *oriC*, near *dif. TerA*–*TerJ* denote polar *Ter*/Tus barriers that arrest replication forks in a directional manner, blocking forks approaching from one side only (black arrows). See text for further details. (**B**) Model for the role of RecG in preventing reverse replication restart during DSE repair (from [37]). This model implies that processing of a DSE by RecBCD may generate an intermediate in which the 5′-ended strand is longer than the 3′-ended strand (see [37] for possible scenarios based on two model for DSE processing by RecBCD [38, 39]). This configuration permits formation of a D-loop in which the invading 3′ end is not properly configured for the correct mode of PriA binding—PriA being the first primosomal protein that acts with additional factors in a cascade culminating in loading of the replicative helicase DnaB, thereby enabling replication restart [28]. RecG-mediated remodeling of the D-loop would convert this structure into an appropriate substrate for the correct PriA binding mode and subsequent DnaB loading, promoting replication restart in the correct orientation (left). In the absence of RecG, PriA may bind in an alternative configuration that directs DnaB loading onto the nascent lagging strand, resulting in reverse replication restart (re-replication; right).

In *E. coli*, bidirectional replication of the circular chromosome begins at *oriC* and terminates in the opposing Ter region, where the two converging forks meet. Resolution of chromosome dimers by XerCD and the final decatenation step by topo IV both occur at the *dif* site, located at the center of Ter [26]. To confine replication termination to this region, forks are arrested by Tus proteins bound to polar *Ter* sequences (10 *Ter*/Tus barriers, *TerA*–*TerJ*; Fig. 1A) [26]. *Ter* sites on the left and right chromosome arms block clockwise- and counterclockwise-moving forks, respectively. When replication initiates exclusively from *oriC* and both forks progress at equal rates without interruption, they merge at the point opposite *oriC*, near *TerC* [26, 27]. However, replication forks may be arrested or slowed for various reasons; for example, fork breakage occurs in ∼18% of cells during each generation [28]. Fork fusion can occasionally trigger over-replication, which is detectable by marker frequency analysis (MFA) of genomic DNA using next-generation sequencing (NGS) as a small Ter peak bounded by *TerA* and *TerB*, as observed in mutants lacking the SbcD, Exo I, Exo VII, or RecJ nucleases ([29, 30], and see below for the much higher Ter peak in *recG* mutants). A similar small Ter peak is observed in *topA* null mutants and is attributed to randomly distributed R-loops that generate replication forks subsequently arrested at *TerA* or *TerB* [31, 32]. A higher Ter peak is detected in *rnhA* (RNase HI) mutants and likely reflects replication initiated from stable R-loops (*oriKs*), some of which may reside within the Ter region; the resulting replication forks also terminate at *TerA* or *TerB* [31, 33–36].

RecG, a helicase that remodels branched DNA structures such as replication forks and D-loops by translocating on double-stranded DNA [40, 41], also helps prevent over-replication during convergent replication-fork fusion [42–44]. Unlike other termination mutants, *recG* cells display a markedly elevated Ter DNA peak [37, 43, 44]. Recent studies indicate that this peak does not result from over-replication caused by fork fusion, but rather from RecA-dependent recombination following replication fork collapse at a *Ter*/Tus barrier, which generates a DSE [30, 37]. In the currently proposed model (Fig. 1B, left) [37], the processing of this DSE by the RecBCD complex generates a substrate for RecA-mediated D-loop formation that may lead to replication restart in the wrong orientation (re-replication). The misoriented fork subsequently collapses at the opposite *Ter*/Tus barrier, regenerating a DSE and reinitiating the process. Iterative cycles of such RecA-dependent, misoriented replication—each producing a new DSE—are proposed to drive progressive amplification of the Ter region (the “back-and-forth” model) [37]. Consistent with this model, RecA ChIP-seq in *recG*-deficient cells revealed outward-directed RecA binding at the *TerA* and *TerB* sites, providing strong evidence for the predicted DSEs oriented for replication forks moving from the terminus toward the origin and being blocked at termination sites (ref) [37]. Moreover, re-replication in a *recG* mutant has been observed at a DSB within the *lacZ* locus, distant from the Ter region, demonstrating that replication in the incorrect orientation can occur following D-loop formation at any chromosomal DSE [37]. In this context, RecG functions to prevent this outcome by remodeling the D-loop intermediate (Fig. 1B, right).

Another situation in which a pronounced Ter-region amplification has been detected by MFA occurs when the *topB* gene is deleted in a *topA* mutant [31, 45]. Recent findings indicate that this elevated Ter peak in *topA topB* mutants arises from replication forks arrested at *Ter*/Tus barriers, which can trigger replication in the incorrect orientation [45]—similar to what is observed in *recG* mutants. Supporting this view, the Ter peak disappears when *tus* is deleted in a *topA topB* background, and inversion of the *TerB*/Tus barrier relocates the amplified region to the interval between *TerB* and *TerG* rather than between *TerB* and *TerA* [45]. Despite these observations, it remains unclear whether Ter DNA amplification proceeds through the same mechanism in *topA topB* and *recG* mutants and, if so, whether topo III and RecG function within a shared pathway to prevent such events. Here, we present results indicating that topo III cooperates with RecG, but not RecQ, to prevent reverse replication from D-loops following RecA-dependent DSE repair. In doing so, topo III limits Ter DNA amplification and facilitates completion of chromosome replication.

## Materials and methods

### Bacterial strains and plasmids

The bacterial strains used in this study are listed in Supplementary Table S1. All *E. coli* K12 strains and their derivatives were grown in liquid LB (Luria-Bertani) medium (Lennox formula) or on LB-Agar plates, with overnight incubation at 37°C, except for the bacterial strains containing pCP20 thermosensitive plasmid, which were grown at 30°C. Cell suspensions were prepared, and aliquots were adjusted to an OD_600_ of 0.01 in fresh LB medium. The cells were grown to log phase at the indicated temperature and up to the indicated OD_600_, prior to experimentation as described below. Antibiotics were supplemented at the following concentrations: ampicillin, 50 µg/ml, chloramphenicol, 15 µg/ml, kanamycin, 20 µg/ml, tetracycline, 10 µg/ml, and spectinomycin, 25 µg/ml. Bacterial strains were constructed by transduction with P1 *vir* as described previously [46]. When required, kanamycin and chloramphenicol resistance cassettes were excised using the pCP20 plasmid, as described [47]. Polymerase chain reaction (PCR) with appropriate oligonucleotides and nucleotide sequencing, when required, were performed to confirm the transfer of the expected alleles in the selected transductants. The plasmid pEAW915, a pACYC184-derivative, carries Super-Glo GFP under control of the SOS-inducible P*recN* promoter [48]. The plasmid pET11-*parCE* produces a ParEC fusion protein that was shown to be active as a topo IV both *in vitro* and *in vivo* [31, 45, 50].

### SOS response assay

The pEAW915 plasmid was introduced into our strains by transformation. The bacterial strains were cultivated at 30°C for 270, 330 and 390 minutes, after which an aliquot of each sample was collected and fixed with 4% paraformaldehyde (PFA) for analysis by flow cytometry. The average fluorescence intensity of GFP per cell was monitored over time, along with the percentage of cells exhibiting an SOS response, using a BD FACSymphony A1 cytometer (BD Life Sciences, USA). About 20 000 events were analyzed per sample, and the results were analyzed using FlowJo software (BD Life Sciences, USA).

### TUNEL assay

DNA breaks were quantified using the terminal deoxynucleotidyl transferase dUTP nick end-labeling (TUNEL) assay. Experimental strains were grown in LB medium at 37°C until mid-log phase, OD_600_ ≃ 0.4, after which the temperature was shifted to 30°C to ensure full manifestation of *topA topB* phenotypes carrying the *gyrBts* allele (see the first section of the ‘Results’ section for the rational for using the *gyrBts* allele). After 2 h of incubation at 30°C, aliquots of the cultures were collected and fixed by the addition of cold 70% ethanol. For positive control, ciprofloxacin (50 ng/ml) was immediately added after the transfer at 30°C and incubated for 180 min prior to the cell fixation. DNA breaks were labeled using the Click-iT™ TUNEL Alexa Fluor™ 488 Imaging Assay Kit (Invitrogen, Thermo Fisher Scientific, USA) according to the manufacturer’s protocol. Following labeling, samples were analyzed by flow cytometry using a BD FACSymphony A1 (BD Life Sciences, USA), with the appropriate settings for Alexa Fluor^TM^ 488 fluorescence. A minimum of 50 000 events were collected per sample. Doublets and aggregates were excluded by appropriate gating. Data was analyzed using FlowJo software (BD Life Sciences, USA). The fluorescence intensity distribution was used to quantify the extent of DNA damage across different strains. The percentage of TUNEL-positive cells was determined by setting thresholds based on unlabeled controls, enabling comparative assessment of DNA damage levels.

### DSEs detection

DSEs were quantified by counting Gam-GFP fluorescent foci as described previously (Gam protein binds to DSEs) [49]. In this system, the *gam-gfp* fusion gene is under the control of the P_N25tetO_ promoter inducible by doxycycline. Strains expressing Gam-GFP were cultivated in LB medium at 37°C until reaching an OD_600_ of 0.4. Doxycycline (200 ng/ml) was then added, and the cells were incubated at 37°C for 60 min to ensure full induction of Gam-GFP expression. The cultures were then shifted to 30°C and incubated for an additional 120 min. As a positive control, ciprofloxacin (50 ng/ml) was added after 60 min of incubation with doxycycline at 37°C and the cells were transferred at 30°C for an additional 180-min incubation. Cells were recovered by centrifugation, washed twice with phosphate-buffered saline (PBS) and fixed with 4% PFA for fluorescence microscopy analysis. The samples were diluted in PBS to achieve an appropriate cell concentration for imaging. Then, 1 μl of each sample was spread on a coverslip pre-treated with poly-L-lysine and allowed to dry at room temperature. The coverslips were then mounted on microscope slides and sealed. Imaging was conducted using a Nikon Eclipse Ti2-E inverted microscope equipped with a Plan Apochromat 1.45 NA oil immersion 100× phase contrast objective and a GFP filter set for epifluorescence imaging, along with a Hamamatsu ORCA Flash 4.0 V2 Digital CMOS Camera C11440-22CU. Foci counting was performed manually and by using ImageJ software.

### DNA staining and visualization

For visualization of cellular DNA content, cells were grown at 30°C to an OD₆₀₀ of 0.8. One ml of culture was harvested, fixed with 4% PFA, and 1 µl was deposited onto a poly-L-lysine–coated coverslip. After air drying, 1 µl of SYTO™ 16 green fluorescent nucleic acid stain (1:100 dilution in ddH₂O; Invitrogen, Thermo Fisher Scientific, USA) was applied directly to the cells. Imaging was performed using a Nikon Eclipse Ti2-E system, as described above.

### Detection of DnaA-independent replication

DnaA-independent replication (Stable DNA Replication, SDR) was detected and monitored by ethynyl deoxyuridine (Edu) incorporation and click-labeling using Click-It^®^ Alexa Fluor 448^®^ Imaging kit (Invitrogen by Thermo Fisher Scientific, USA) as previously described [45, 51, 52]. Overnight cultures were diluted to an OD_600_ of 0.01 and grown to an OD_600_ of 0.3 at 30°C. An aliquot of cells was used for EdU incorporation over 60 min to detect ongoing replication in log-phase cells. For SDR, the log-phase cells were treated with spectinomycin (400 µg/ml) or rifampicin (300 µg/ml) for 120 min to allow the termination of replication rounds originating from *oriC* prior to EdU incorporation. Following click-labeling with the Alexa Fluor^®^ 488 dye, EdU incorporation was recorded using a BD FACSymphony A1 cytometer (BD Life Sciences, USA), and histograms were obtained using FlowJo software (BD Life Sciences, USA).

### Marker frequency analysis

MFA by NGS was performed essentially as previously described [45]. Cells were grown at 30°C to an OD_600_ of 0.4. An aliquot of 15 ml of cell culture was transferred to a tube filled with ice for genomic DNA extraction. Spectinomycin (400 μg/ml) was added to the remaining culture, and the cells were incubated for an additional 2 h at the same temperature. An aliquot of 15 ml of cell culture was then taken and treated as above. Cells were pelleted by centrifugation, and genomic DNA was isolated using a GenElute^TM^ Bacterial Genomic DNA Kit (Sigma–Aldrich, Germany) following the manufacturer’s guidelines, with an extended proteinase K treatment of 2 h. Sequencing was performed using the Illumina NextSeq 2000 platform (Illumina Innovative Technologies, San Diego, USA) to ascertain sequence copy number (Genomics Platform of IRIC, Institute for Research in Immunology and Cancer, Montréal, Canada). Bioinformatics analysis was undertaken at the IRIC’s Bioinformatics Platform. Each sample generated ∼13–22 million sequencing reads. Sequences were trimmed for sequencing adapters and low quality 3′ bases using Trimmomatic version 0.35 [53] and aligned to the reference *E. coli* K12 W3110 genome (assembly AP009048.1) using BWA mem version 0.7.12 [54]. After removal of PCR duplicates and secondary alignments, coverage was computed along the whole genome and normalized to log_2_(RPM + 1), where RPM stands for reads per million mapped reads, to account for sequencing depth. The number of reads was normalized against the isogenic stationary phase wild-type control [MM62 (RFM443) stat (SRR7695728), SRA project PRJNA486133] to consider differences in read depth across the genome. Loess regression curves were generated with loess-span parameters set to 0.1.

### DNA content measurement in rifampicin run-out experiments.

To assess replication completion in our strains, cellular DNA content was quantified by flow cytometry after rifampicin run-out, as previously described [46]. Strains were grown in LB medium at 30°C up to an OD_600_ of 0.3. For every experiment, a wild-type control was performed. Rifampicin (300 μg/ml) and cephalexin (10 μg/ml) were then added to inhibit the initiation of new rounds of replication from *oriC* and to halt cell division, respectively. Aliquots of the culture were collected at defined time points (0, 30, 60, 90, and 120 min post-treatment) and immediately fixed in 70% ethanol. The samples were stored at 4°C for about 18 h to ensure complete fixation and then washed twice with 1× PBS, pH 7.4, followed by the treatment with 200 μg/ml of RNase A (Sigma–Aldrich, USA) and staining with 5 μM of SYTO™ 16 green fluorescent nucleic acid stain (Invitrogen, Thermo Fisher Scientific, USA) for 30 min in the darkness. Samples were analyzed using a BD FACSymphony A1 cytometer (BD Life Sciences, USA), with the appropriate settings for SYTO 16 fluorescence. A minimum of 20 000 events were recorded per sample, and doublets or aggregates were excluded using forward and side scatter pulse geometry gating. Data were analyzed using FlowJo software (BD Life Sciences, USA), allowing quantitative comparison of DNA content distributions across strains and time points.

### Statistical analysis

Experiments were conducted in multiple replicates, and the final data were displayed as the mean ± standard error of the mean. Statistical differences among samples were evaluated using one-way analysis of variance followed by Dunnett’s multiple comparisons test. The student’s unpaired *t*-test with Welch’s correction was employed to compare two groups. Statistical significance was considered at *P* <.05. All statistical analyses were performed using GraphPad Prism version 10 (GraphPad Software, MA, USA).

## Results

### The induction of the SOS response in topA topB null cells is largely Tus-dependent.

To investigate DNA damage in *topA topB* null cells, we first monitored activation of the SOS response, the canonical DNA damage response pathway in bacteria. For this purpose, we introduced the low-copy number plasmid pEAW915, which carries Super-Glo GFP under control of the SOS-inducible P*recN* promoter [48], into a set of isogenic strains. All these strains, but the wild-type, carry the *gyrBts* allele. The *gyrBts* allele encodes a partially defective GyrB protein at 37°C, reducing gyrase-mediated negative supercoiling. This suppression allows strains carrying a *topA* null mutation to grow more efficiently at 37°C than at 30°C [55]. At 30°C, gyrase activity is restored, and the phenotypes associated with *topA* and *topA topB* null mutations fully manifest—slow growth, excessive negative supercoiling, elevated R-loop formation, unregulated replication (most prominently in *topA topB* cells), and a high-level of Ter DNA amplification (in *topA topB* null cells) [31, 45, 55, 56]. Therefore, unless otherwise indicated, the data presented in this paper were obtained from strains grown at 30°C. In our studies related to DNA damage we used ciprofloxacin as a positive control. Ciprofloxacin, a fluoroquinolone antibiotic, targets DNA gyrase and topoisomerase IV, trapping these enzymes on the bacterial chromosome and inducing DSBs [57].

**Figure 2.**
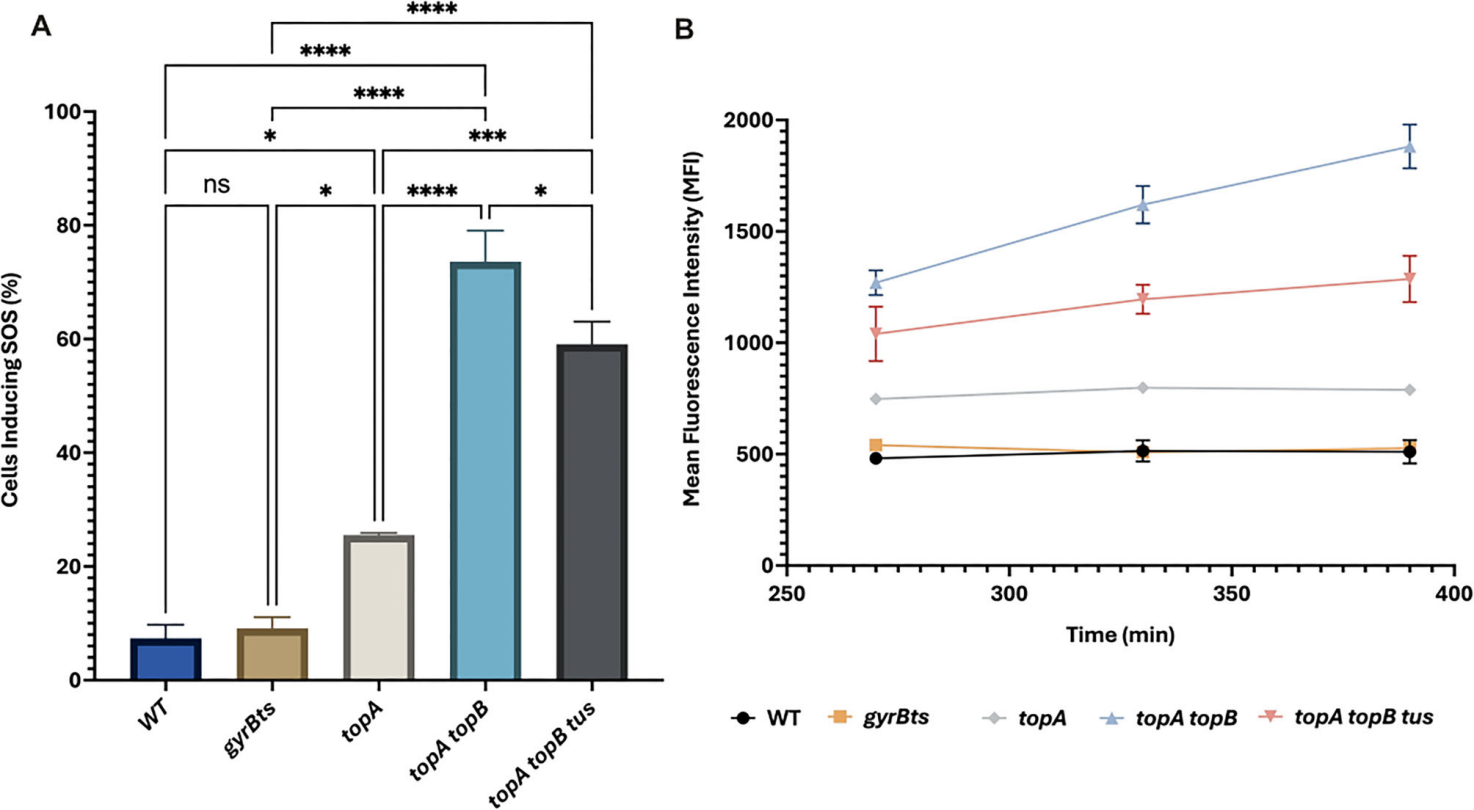
Tus-dependent induction of the SOS response in *topA topB* null cells. RFM443 (WT), MM63 (*gyrBts*), JB206 (*topA20::*Tn*10 gyrBts*), JB395 (*topA20::*Tn*10 ΔtopB gyrBts*), and JB260 (*topA20::*Tn*10 ΔtopB Δtus gyrBts*) cells carrying the SOS-reporter plasmid pEAW915 were grown at 30°C and prepared for flow cytometry analysis as described in the ‘Materials and methods’ section. (**A**) Percentage of cells inducing the SOS-response [data corresponding to the last time point shown in panel (B)]. (**B**) Average SOS-induction level (mean fluorescence intensity) per cell over time.

Induction of the SOS response was quantified by flow cytometry as described in the ‘Materials and methods’ section. First, a positive control experiment using ciprofloxacin confirmed that induction of the SOS response could be reliably detected by flow cytometry in strains carrying pEAW915 (Supplementary Fig. S1). As shown in Fig. 2, RFM443 (wild type) and RFM445 (*gyrBts*) exhibited nearly identical basal values: ∼6–8% of cells expressed the reporter fusion (A), with an average fluorescence of ∼500 per cell (B). These results indicate that the *gyrBts* allele alone did not influence SOS induction under our assay conditions. Deleting *topA* (strain JB206) resulted in a modest increase in SOS expression: ∼25% of cells expressed the fusion at an average value slightly above 700. This low-level induction remained stable over time. In contrast, when both type IA topoisomerases were absent (JB395, *topA topB*), the SOS response was significantly activated: ∼75% of cells were induced, with average fluorescence exceeding 1800 by the end of the experiment. Notably, SOS induction increased progressively over time consistent with ongoing accumulation of DNA damage in *topA topB* cells at 30°C. When the *tus* gene was deleted in the *topA topB* background (JB260, *topA topB tus*), SOS induction was reduced to ∼60% of cells, with an average value of ∼1250 at the end of the experiment. Importantly, unlike in *topA topB* cells, the level of induction only slightly increased over time. Together, these observations show that DNA damage accumulate significantly over time only when Tus is present, indicating that most of the DNA damage accumulating in *topA topB* mutants arises from replication problems linked to *Ter*/Tus fork-barriers.

### TUNEL analysis indicates that DNA break accumulation in topA topB null cells is largely Tus-dependent

Because *topA topB* null cells showed significant induction of the SOS response, we next examined whether they accumulate DNA breaks. To do this, we used a TUNEL assay (Terminal deoxynucleotidyl transferase dUTP Nick-End Labeling), in which TdT incorporates EdU, a thymidine analog, at 3′-OH DNA ends. These ends occur in gapped DNA as well as at single-strand breaks or DSBs. Alexa Fluor 488 is then conjugated to EdU via click chemistry, and EdU incorporation is quantified by flow cytometry. Experiments were performed as described in the ‘Materials and methods’ section.

**Figure 3.**
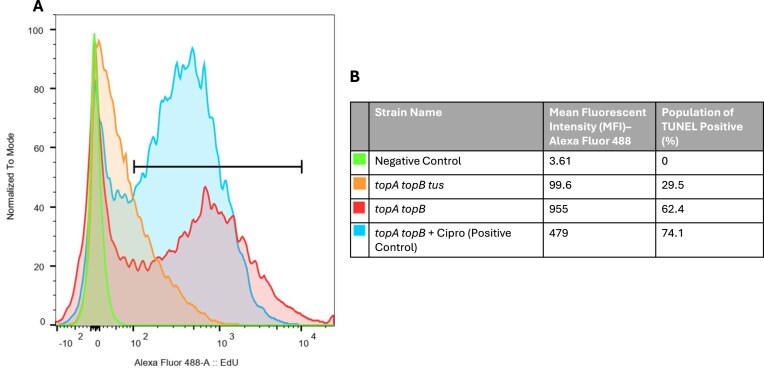
The TUNEL assay reveals a high level of Tus-dependent DNA breaks accumulation in *topA topB* null cells. JB395 (*topA20::*Tn*10 ΔtopB gyrBts*, + and – ciprofloaxacin) and JB260 (*topA20::*Tn*10 ΔtopB Δtus gyrBts*) cells were grown at 30°C and prepared for the TUNEL assay as described in the ‘Materials and methods’ section. (**A**) Histogram showing the distribution of DNA damage intensity (Alexa Fluor™ 488 labeling intensity) across the indicated strains. The black line denotes the TUNEL-positive region. (**B**) Mean DNA damage intensity and percentage of cells exhibiting DNA damage. JB395 cells not treated with TdT were used as the negative control.

As shown in Fig. 3, *topA topB* and *topA topB tus* strains displayed 62.4% and 29.5% TUNEL-positive cells (A), with mean fluorescence intensities of 995 and 99, respectively (B). The positive ciprofloxacin control yielded 74.1% TUNEL-positive cells with an average intensity of 479. These results demonstrate a high level of DNA breaks in *topA topB* cells that is strongly dependent on Tus and therefore arises from replication problems linked to *Ter*/Tus fork barriers, consistent with the SOS assay results.

### A Gam–GFP fusion demonstrates a pronounced accumulation of DSEs in topA topB null cells that requires both Tus and RecA

The TUNEL assay revealed an accumulation of DNA breaks but cannot distinguish among gaps, ssDNA ends, or DSEs. The re-replication model leading to Ter DNA amplification involves the formation of DSEs at *Ter*/Tus barriers, which are processed by RecBCD to generate the substrate for RecA. To directly quantify DSE accumulation, we used a previously developed system based on the bacteriophage Mu DSE-binding protein Gam fused to GFP [49]. In this system, a Mu *gam-gfp* fusion gene in the *E. coli* chromosome is controlled by the doxycycline-inducible PN25*tetO* promoter. In all experiments, two controls were included: a negative control without doxycycline and a positive control with doxycycline and ciprofloxacin. The positive control, expected to produce many cells with foci, was included to verify expression of the *gam-gfp* fusion gene in our strains and to establish the appearance of foci in our imaging system. Experiments were performed as described in the ‘Materials and methods’ section.

**Figure 4.**
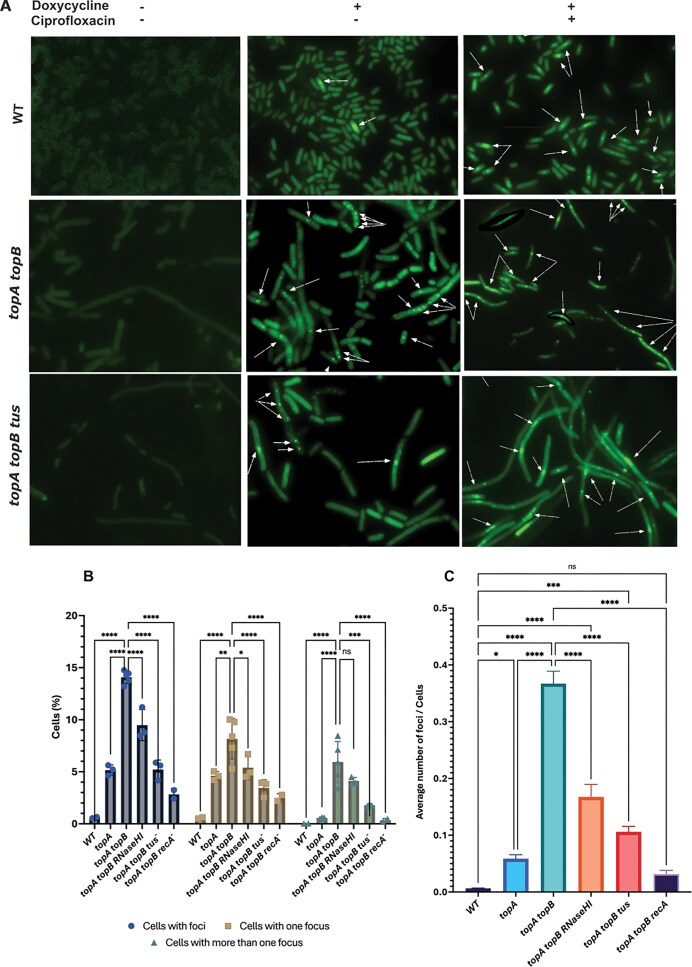
A Gam-GFP reporter fusion reveals high levels of Tus- and RecA-dependent DSEs in *topA topB* null cells. MM68 (WT), MM69 (*ΔtopA gyrBts*), AD95 (*topA20::*Tn*10 ΔtopB gyrBts*), AD85 (*topA20::*Tn*10 ΔtopB Δtus gyrBts*), AD294 (*topA20::*Tn*10 ΔtopB ΔrecA1921::spc gyrBts*), and AD45 (*topA20::*Tn*10 ΔtopB gyrBts*/pSK760) strains carrying a Gam-GFP reporter fusion were grown at 30°C and prepared for fluorescence microscopy for Gam-GFP foci observation and quantification, as described in the ‘Materials and methods’ section. The total number of cells analyzed and the number of independent experiments for each strain were as follows: WT, 5762 cells (*n* = 2); *topA*, 9142 (*n* = 3); *topA topB*, 5976 (*n* = 5); *topA topB tus*, 4873 (*n* = 3); *topA topB recA*, 3811 (*n* = 2); and *topA topB/*pSK760, 6192 (*n* = 3). (**A**) Representative images of fluorescence microscopy of WT, *topA topB* and *topA topB tus* cells. From left to right, − doxycycline (negative control), + doxycycline (experimental sample), and + doxycycline + ciprofloxacin (positive control). White arrows show examples of foci. White lines represent 10 µm. (**B**) Graphical representation of the percentage of cells containing foci, cells with a single focus and those with multiple foci. (**C**) Graphical representation of the mean number of foci per cell.

Figure 4A shows example images used to quantify Gam–GFP foci, with arrows indicating examples of foci. As evident, no foci are detected in the negative controls, whereas a majority of cells contain foci following ciprofloxacin treatment. Wild-type cells exhibit very few foci, as expected, while *topA topB* null cells show a significant increase in the number of foci. These observations are confirmed by the quantification shown in Fig. 4B. Only 0.59% of wild-type cells contained at least one focus, with 0.035% containing more than one. In *topA* null cells, 5.2% of cells contained at least one focus and 0.55% contained more than one. In *topA topB* null cells, these values increased to 14.1% and 5.9%, respectively. These results are consistent with the significant induction of the SOS response observed in *topA* mutants only in the absence of *topB*, as well as with the much higher level of Ter DNA amplification in *topA topB* null cells compared with *topA* null cells ([ref. (31)] and see below). Deletion of *tus* in *topA topB* null cells markedly reduced the proportion of cells containing at least one focus from 14.1% to 5.2%, and those containing more than one focus from 5.9% to 1.8%. We consider the observation that 14.1% of *topA topB* null cells contain at least one DSB to be highly significant, given the major consequences of DSBs for genomic instability, including their potential to promote antibiotic resistance in bacteria, and the nearly 25-fold higher frequency observed relative to wild-type cells.

During microscopy analysis, we observed numerous filamentous *topA topB* cells, many containing more than two foci (e.g. Fig. 4A), whereas such elongated cells were rarely observed in *topA* null cells. To better illustrate differences in DSB levels, we quantified the total number of foci per cell and expressed the data as the average number of foci per cell (Fig. 4C). This yielded mean values of 0.37 and 0.050 foci per cell for *topA topB* and *topA* null cells, respectively, corresponding to a 7.4-fold difference between these strains, compared with a 2.7-fold difference when the data were expressed as the total number of cells with foci. These results further illustrate the strong effect of *topB* deletion on DSB formation and SOS induction leading to filamentation in *topA* null cells. Consistent with the distributions shown in Fig. 4B, mean values of 0.37 and 0.11 foci per cell were obtained for *topA topB* and *topA topB tus* null cells, respectively. These results demonstrate that the Tus-dependent high levels of SOS induction and DNA break accumulation in *topA topB* null cells are largely attributable to the formation of DSEs at *Ter*/Tus replication-fork barriers.

As a control, the data shown in Supplementary Fig. S2 demonstrate, as expected, that these DSEs are largely dependent on restored gyrase activity, as the number of Gam–GFP foci decreased by more than threefold when the type IA topo mutant cells (carrying the *gyrBts* allele) were grown at 37°C rather than 30°C. Interestingly, deletion of *tus* in *topA* null mutants led to a slight increase in the average number of foci per cell, from 0.07 to 0.11 (Supplementary Fig. S3, *topA* versus *topA tus*). This value is very similar to that observed in *topA topB tus* cells (≈0.11; Fig. 4C), indicating the formation of Tus-independent DSEs as a consequence of *topA* deficiency. Furthermore, in agreement with previous observations that RNase HI overproduction reduced the Ter peak height in *topA topB* null cells ([31)] and see below), our results show that RNase HI overproduction also decreased both the proportion of cells with foci (Fig. 4B) and the average number of foci per cell (Fig. 4C).

According to the back-and-forth re-replication model, RecA mediates the assembly of a new replication fork at one *Ter*/Tus barrier, which in turn leads to the formation of a DSE at the opposing *Ter*/Tus barrier. Consequently, a substantial fraction of the DSEs detected in *topA topB* null cells is expected to be RecA-dependent. Consistent with this prediction, Fig. 4B shows that deletion of *recA* reduced the proportion of *topA topB* null cells containing at least one Gam–GFP focus from 14.1% to 2.8%. Moreover, this value is lower than the 5.2% observed in *topA topB tus* null cells, suggesting the additional presence of RecA-dependent DSEs that are independent of *Ter*/Tus barriers in *topA topB* null cells.

### Ter DNA amplification in topA topB null mutants is RecA-dependent

If Ter DNA amplification in *topA topB* null cells arises from RecA-dependent DSEs, then MFA by NGS should reveal no Ter DNA peak in *topA topB recA* cells. Furthermore, ongoing RecA-dependent amplification should be accompanied by detectable RecA-dependent replication. Previously, qPCR (quantitative polymerase chain reaction) assays suggested that no Ter DNA amplification occurs in *topA topB* null mutants in the absence of *recA* [31]. However, qPCR is less sensitive than MFA and does not exclude the possibility that lower levels of Ter DNA amplification still occur in the absence of *recA*. MFA by NGS was performed as described in the ‘Materials and methods’ section. Supplementary Fig. S4 shows MFA profiles of control strains—wild-type (RFM443), *gyrBts* (RFM445), and *topB gyrBts* (VU409)—with or without spectinomycin (Spc) treatment for 2 h. Spc inhibits replication initiation from *oriC* while allowing ongoing replication to finish and unregulated non-*oriC*-dependent initiation (e.g. R-loop- or RecA-dependent) to continue. All strains show similar profiles, with an *oriC* peak and a Ter trough, and a flat profile after Spc treatment, indicating completion of replication.

**Figure 5.**
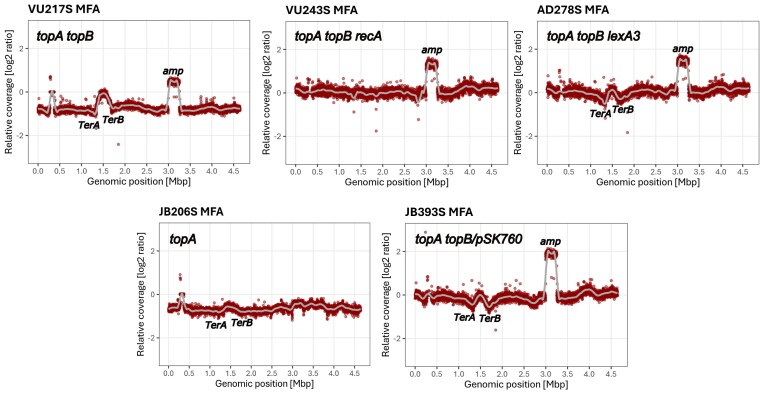
MFA demonstrates that Ter DNA amplification is strictly RecA dependent but only partially dependent on R-loop formation and SOS induction. MFA by NGS of genomic DNA extracted from VU217 (*ΔtopA ΔtopB gyrBts*), VU243 (*ΔtopA ΔtopB ΔrecA306 srlR301::Tn10 gyrBts*), AD278 (*ΔtopA ΔtopB lexA3 gyrBts*) JB206 (*topA20::*Tn*10 gyrBts*) and JB393 (*topA20::*Tn*10 ΔtopB gyrBts*/pSK760). Cells were grown at 30°C to log phase and treated with spectinomycin for 2 h for genomic DNA extraction as described in the ‘Materials and methods’ section. pSK760 carries the wild-type *rnhA* gene to overproduce RNase HI. The number of nucleotide sequence reads, expressed as log₂ values normalized to a stationary-phase wild-type control, is plotted as a function of chromosomal position. Only the chromosomal regions relevant to this study are shown, including the *TerA* and *TerB* barriers and the amplified *parC–parE* region (*amp*). The gray line represents the loess regression curve. The gap at position around 0.3 corresponds to the *Δ(codB-lacI)3* deletion carried by the strains used in this work (do not take into account the loess curve in this area). The strain name is indicated above each MFA diagram and is followed by the letter S, denoting spectinomycin addition during growth.

Figure 5 (top) shows MFA profiles of three isogenic *topA topB* null strains treated with spectinomycin and carrying or not a *recA* deletion or the *lexA3* mutation. As shown before, the MFA profile of the *topA topB* strain (VU217) is characterized by a prominent Ter peak on the left (within the *TerA*–*TerB* interval) and amplification of a DNA region on the right encompassing the *parC* and *parE* genes (encoding the topo IV subunits), enabling topo IV overproduction. *topA topB* null cells in both *E. coli* and *Bacillus subtilis* maintain amplification of the topo IV genes for viability [31, 58]. Figure 5 (top) also shows that deletion of *recA* in *topA topB* null cells completely abolishes the Ter DNA peak (VU217, *topA topB* versus VU243, *topA topB recA*). Because RecA mediates both homologous recombination and SOS induction, we tested whether Ter DNA amplification requires the SOS function by using the non-inducible *lexA3* allele. In *topA topB lexA3* cells, the Ter DNA peak remained evident, albeit reduced compared with *topA topB* cells (VU217, *topA topB* versus AD278, *topA topB lexA3*). These results indicate that Ter DNA amplification depends on the recombination function of RecA but does not require its SOS-induction activity. The reduced Ter peak in *lexA3* cells likely reflects lower RecA levels, as *recA* is partially SOS-regulated and SOS is induced in *topA topB* cells. Figure 5 (bottom) shows very low Ter DNA peaks and topo IV gene amplification in *topA* null cells, as well as a reduction in Ter DNA amplification in *topA topB* null cells upon RNase HI overproduction (JB393, *topA topB*/pSK760), as previously reported [31].

RecA loading during DNA repair occurs via distinct pathways: RecBCD at DSEs and RecFOR at single-strand DNA gaps [59]. If Ter DNA amplification in *topA topB* null cells originates from DSEs, it should therefore depend on RecBCD but not on RecFOR. Direct testing of RecBCD involvement was not possible because *topA topB recB* mutants could not be constructed. In contrast, a *topA topB recO* mutants could be constructed and Ter DNA amplification was still observed (Supplementary Fig. S5), demonstrating that the RecFOR pathway is dispensable.

### RecA-dependent replication is activated in topA topB null mutants

As noted above, protein synthesis inhibitors block replication initiation at *oriC*. Under these conditions, two alternative replication initiation mechanisms—collectively termed stable DNA replication (SDR)—can occur [60, 61]. Inducible SDR (iSDR) is SOS-inducible and requires RecA and RecBCD, whereas constitutive SDR (cSDR) is observed in *rnhA* mutants, involves R-loops, and is sensitive to rifampicin (rifampicin also blocks replication initiation at *oriC*) [60, 61]. cSDR also occurs in *topA* and *topA topB* null mutants [52]. Our data may suggest that both cSDR and iSDR operate in *topA topB* mutants, with iSDR occurring in the Ter region and contributing to Ter DNA amplification. To test this hypothesis, we used our SDR assay based on incorporation of the thymidine analog EdU, which is detected by Alexa Fluor 488 via click chemistry, as described in the ‘Materials and methods’ section. For each strain, the following conditions were tested: no EdU (to assess nonspecific Alexa Fluor 488 binding), EdU without inhibitors (positive replication control), and EdU following a 2-h treatment with spectinomycin or rifampicin.

**Figure 6.**
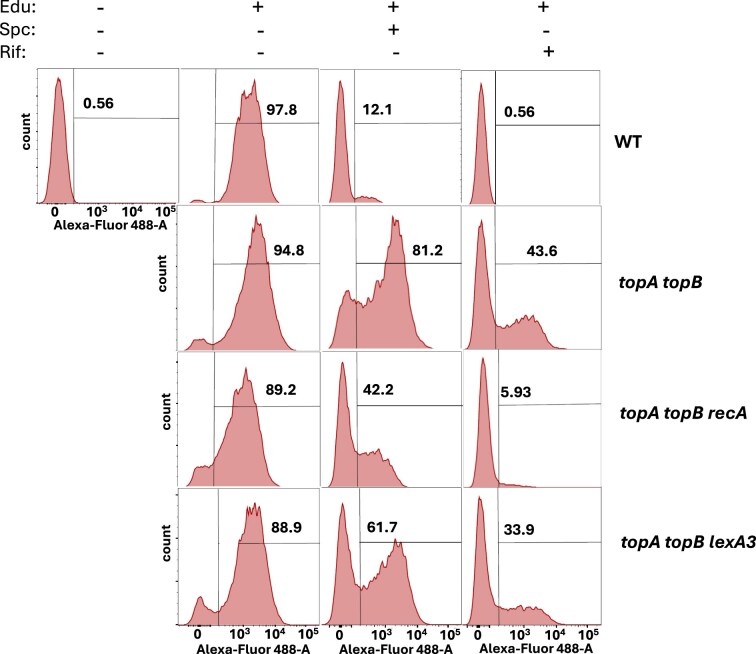
Both cSDR and iSDR are activated in *topA topB* null cells. Flow cytometry was used to detect SDR in RFM443 (wild type), VU217 (*ΔtopA ΔtopB gyrBts*), VU243 (*ΔtopA ΔtopB ΔrecA306 srlR301::Tn10 gyrBts*) and AD278 (*ΔtopA ΔtopB lexA3 gyrBts*) cells grown at 30°C as described in the ‘Materials and methods’ section. For the wild-type strain, the left panel represents a no-EdU control. In all other panels, as indicated, EdU was added after treatment with, from left to right, no antibiotic, spectinomycin, or rifampicin. Numbers in the upper right of each panel indicate the percentage of cells incorporating EdU, reflecting ongoing DNA replication.

In Fig. 6 (top), the left panel shows the no-EdU control, in which the peak corresponds to nonspecific dye binding. This control was used to define the vertical reference line separating nonspecific signal (left) from EdU incorporation (right). The second panel shows cells incorporating EdU in the absence of inhibitors, with the replication-associated peak located to the right of the vertical line. The third and fourth panels show cells treated for 2 h with spectinomycin or rifampicin, respectively, prior to EdU addition. In the case of RFM443, the result demonstrates the absence of SDR (very low level in spc treated cells and nothing detected in rif treated cells) in wild-type cells.

In contrast, a high level of SDR was detected in *topA topB* null cells (VU217), approximately half of which corresponds to cSDR, as reflected by 81.2% and 41.6% of cells incorporating EdU following spectinomycin and rifampicin treatment, respectively (Fig. 6, second row). The remaining fraction likely represents iSDR and should therefore be RecA-dependent. Consistent with this prediction, deletion of *recA* in *topA topB* null cells (VU243) reduced EdU incorporation by approximately half in spectinomycin-treated cells (81.2% in VU217 versus 42.2% in VU243), and completely abolished SDR following rifampicin treatment (third row). Thus, roughly half of the SDR observed in *topA topB* null cells is RecA-dependent (iSDR), while the other half is R-loop-dependent (cSDR). It is noteworthy that a similar half-and-half distribution between iSDR and cSDR has been described in *recG* null cells [62]. Consistent with the MFA results, the *lexA3* allele reduced the level of RecA-dependent replication (AD278, *topA topB lexA3*, compared with VU217 and VU243) (fourth row). Altogether, the MFA and SDR results support the back-and-forth model in which RecA-dependent re-replication drives Ter DNA amplification. Our results further predict that Ter DNA amplification persists after spectinomycin addition. This is consistent with the observation that, in *topA topB* null cells, the Ter DNA peak height continues to increase following spectinomycin treatment, as revealed by MFA [45].

### The level of Ter DNA amplification increases significantly upon deleting topB in rnhA cells but not upon deleting topB in recG cells

To date, all evidence implicating topo III in suppressing re-replication within the Ter region has been obtained in cells that also lack topo I. Ter DNA amplification is triggered by replication fork collapse at *Ter*/Tus barriers, a process that is favored when replication forks accumulate at these sites. Such accumulation may occur due to R-loop–initiated replication in *topA* mutants [31, 52] and to replication initiated by fork fusion within the *TerA*–*TerB* interval in *recG* mutants [43]. Replication from R-loops also occurs in *rnhA* mutants and results in a Ter DNA peak [31, 34, 35, 60]. If topo III acts in concert with RecG to prevent re-replication and consequent Ter DNA amplification, then loss of topo III should increase the height of the Ter DNA peak in *rnhA* mutants.

**Figure 7.**
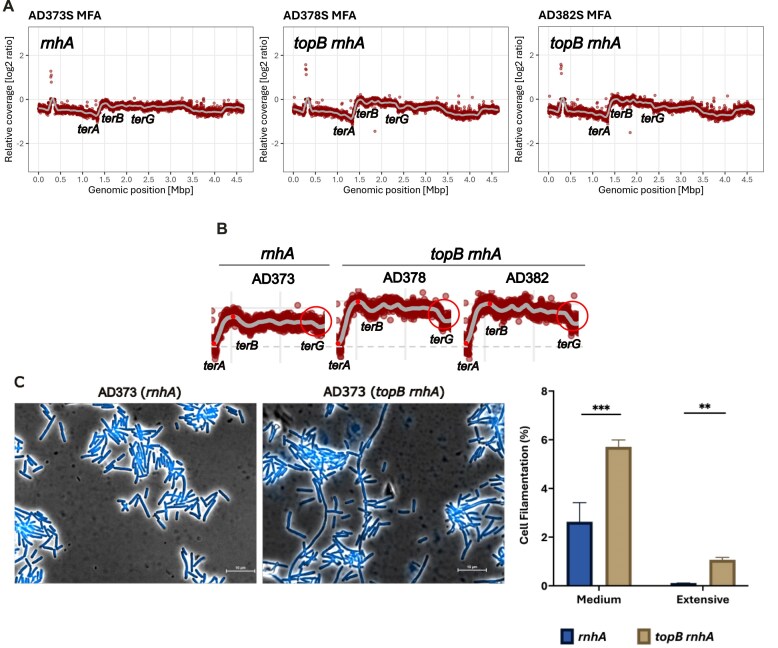
MFA showing that deletion of *topB* increases the level of Ter DNA amplification in a *rnhA* mutant. MFA by NGS of genomic DNA extracted from AD373 (*ΔrnhA::cam gyrBts*) and from two *ΔrnhA::cam ΔtopB gyrBts* independent transductants, AD378 and AD382. Cells were grown at 30°C to log phase and treated with spectinomycin for 2 h for genomic DNA extraction as described in the ‘Materials and methods’ section. The full MFA profiles are shown in panel (A) whereas in panel (B) the MFA profile encompassing the Ter region up to the *TerG* barrier is enlarged to facilitate visualization of the differences between *rnhA* and *rnhA topB* strains. The gray line is the loess regression curve. *TerA, TerB*, and *TerG* barriers are indicated. The region within the red dotted circle highlights a pronounced drop in copy number at the *TerG* barrier in *topB rnhA* cells, which is not observed in *rnhA* cells. The red dots on the loess curves indicate the lower (*TerA*) and higher (top of the Ter peak) copy numbers within the Ter region. (**C**) Microscopy images of SYTO 16 stained AD373 (*rnhA gyrBTs*) and AD378 (*rnhA topB gyrBts*) cells. A total of 2205 (*n* = 2) and 1640 (*n* = 2) cells were analyzed for AD373 and AD378 strains, respectively, to determine the proportion of cells exhibiting moderate or extensive filamentation. The strain name is indicated above each MFA diagram.

Figure 7A and B show that this is indeed the case for the two independent *rnhA topB* clones analyzed by MFA [compare AD373 (*rnhA*) with AD378 and AD382 (*rnhA topB*)]. In panel (B), the MFA profile encompassing the Ter region up to the *TerG* barrier is enlarged to facilitate visualization of the differences between *rnhA* and *rnhA topB* strains. From *TerA* to the peak maximum, deletion of *topB* in *rnhA* cells resulted in an ∼60% increase in copy number. In addition, a pronounced drop in copy number—producing a distinct break in the MFA profile at the *TerG* barrier—was observed in *rnhA topB* cells (AD378 and AD382). This feature may indicate the accumulation of clockwise-moving replication forks—likely initiated from the *oriK*(s) (bidirectional replication) previously identified between *TerB* and *TerG* [31, 34, 35]—at *TerG*, leading to re-replication initiated from *TerG* in the counterclockwise orientation. Replication forks initiated between *TerB* and *TerG* that move counterclockwise [*oriK*(s)], as well as those potentially initiated from *TerG* in the same direction, pass *TerB* and converge at *TerA*, which likely explains the MFA pattern showing a substantially lower copy number at *TerA* compared with *TerB*. Together, these MFA results in *rnhA topB* null cells support a role for topo III, independent of topo I, in preventing re-replication at *Ter*/Tus barriers. Interestingly, the presence of long filamentous cells is more easily observed in *rnhA topB* (AD378) than *rnhA* (AD373) null cells (Fig. 7C).

**Figure 8.**
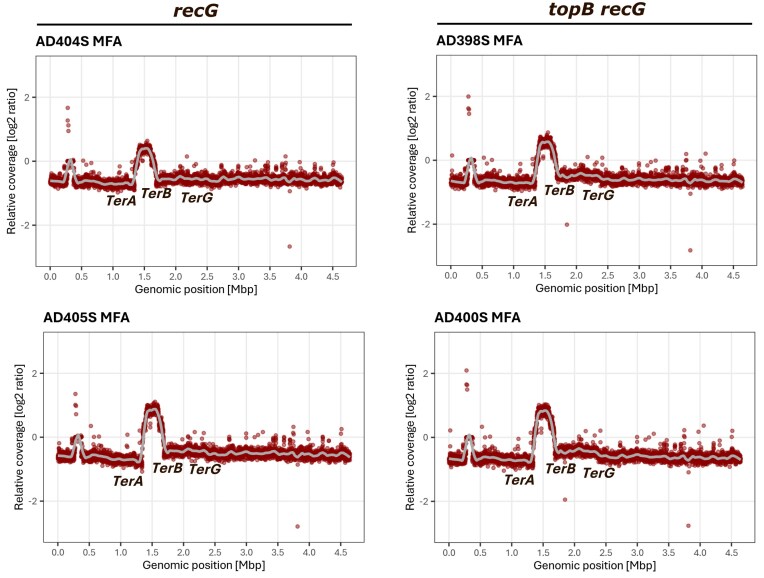
MFA showing that deletion of *topB* does not significantly modify the MFA DNA profile of a *recG* mutant. MFA by NGS of genomic DNA extracted from two independent *ΔrecG gyrBts* transductants, AD404 and AD405 and from two independent *ΔrecG ΔtopB gyrBts* transductants, AD398 and AD400. Cells were grown at 30°C to log phase and treated with spectinomycin for 2 h for genomic DNA extraction as described in the ‘Materials and methods’ section. The gray line is the loess regression curve. *TerA, TerB*, and *TerG* barriers are indicated. The strain name is indicated above each MFA diagram and is followed by the letter S, denoting spectinomycin addition during growth.

Given that two independent *topB recG* clones showed small differences in Ter DNA peak height by MFA (Fig. 8; compare clones AD398 and AD400), we analyzed two independent *recG* clones for comparison. The MFA profiles of the *recG* clones showed similar fluctuations in Ter DNA peak height (Fig. 8; compare clones AD404 and AD405). When the average Ter peak heights were compared between *topB recG* and *recG* strains, no significant differences were observed. Moreover, in contrast to the *rnhA* versus *rnhA topB* comparison, no changes in MFA profiles were detected outside the Ter region. We therefore conclude that deletion of *topB* in *recG* cells does not significantly affect either Ter DNA peak height or the overall MFA pattern. These results support the possibility that topo III acts together with RecG to suppress Ter DNA amplification.

### Evidence for topB cooperation with recG but not recQ

Because the eukaryotic homologs of *E. coli* RecQ—Sgs1 in yeast and BLM in humans—function with topo III during recombination, we asked whether RecQ similarly cooperates with topo III to limit Ter DNA amplification in a *topA* mutant. As shown in Supplementary Fig. S6, this is not the case. In contrast to deletion of *topB*, deletion of *recQ* in a *topA* background did not enhance the low-level Ter DNA peak observed in *topA* cells [compare *topA recQ* (JB553) with *topA* (JB206)]. Moreover, the *topA recQ* strain showed no amplification of topo IV genes, whereas *topA topB* mutants display strong topo IV gene amplification that is required for viability. These results demonstrate that RecQ does not function with topo III to prevent Ter DNA amplification.

**Figure 9.**
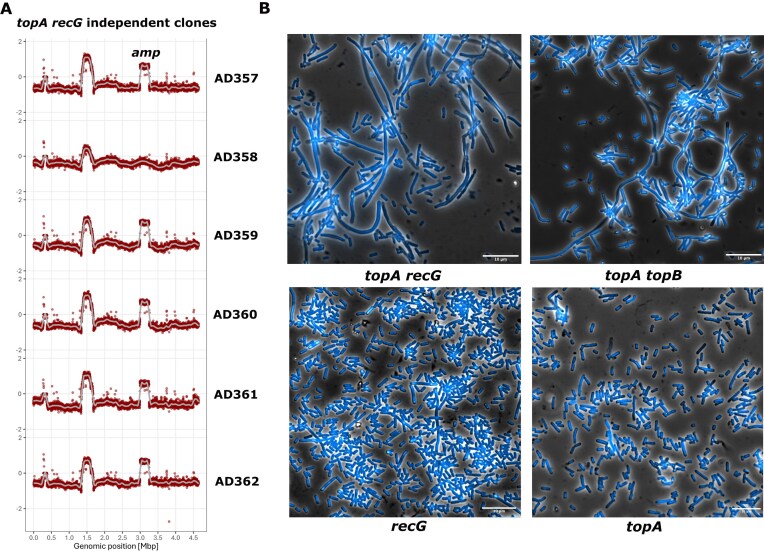
Similar to *topA topB* strains, *topA recG* strains exhibit amplification of topo IV genes and extensive cellular filamentation. (**A**) MFA by NGS of genomic DNA extracted from six independent *ΔrecG* transductants of *topA20::*Tn*10 gyrBts* strain. Cells were grown at 30°C to log phase and treated with spectinomycin for 2 h for genomic DNA extraction as described in the ‘Materials and methods’ section. *amp* indicates the amplified *parC parE* region. (**B**) AD360 (*topA20::*Tn*10 ΔrecG gyrBts*), JB395 (*topA20::*Tn*10 ΔtopB gyrBts*), AD404 (*ΔrecG gyrBts*), and JB206 (*topA20::*Tn*10 gyrBts*) were grown at 30°C to an OD_600_ of 0.8 and prepared for microscopy as described in the ‘Materials and methods’ section. Representative merged images of phase contrast and fluorescence pictures of SYTO-16-stained cells are shown.

So far, our data indicates that RecG, rather than RecQ, functions with topo III. As described above, a conserved feature of *topA topB* null mutants in both *E. coli* and *B. subtilis* is their dependence on high-level amplification of topo IV genes for viability (with one exception in *E. coli*, to be reported elsewhere). To determine whether loss of *recG* creates a similar requirement, we introduced a *recG* null allele into a *topA* strain by transduction and recovered six independent transductants for genomic DNA analysis by MFA. As expected, *recG* single mutants do not exhibit topo IV gene amplification (e.g. Fig. 8, strain AD405). In contrast and mirroring *topA topB* null mutants, five of six of the independent *topA recG* null clones showed high-level amplification of topo IV genes (Fig. 9A). Furthermore, unlike *topA* or *recG* single mutants but similar to *topA topB* mutants, *topA recG* cells formed long filaments (Fig. 9B). Together, these results further support a functional interplay between topo III and RecG in *E. coli*.

Using rifampicin run-out experiments coupled with flow cytometry, we recently identified a severe defect in replication completion in *topA topB* null cells. In these assays, rifampicin and cephalexin are added to early log-phase cultures. Rifampicin blocks new rounds of replication initiation from *oriC* (and from R-loops), thereby allowing ongoing rounds of DNA replication to complete, whereas cephalexin inhibits cell division. Cellular DNA is then stained with SYTO 16 and analyzed by flow cytometry. The resulting DNA content profiles reflect the number of fully replicated chromosomes per cell and, consequently, the number of replication origins activated at the time rifampicin was added. In cells with properly regulated, mass-synchronized replication initiation, discrete 2*n* chromosome DNA content peaks are observed, where *n* corresponds to the number of activated *oriC* at the time of rifampicin addition. In wild-type cells, narrow and well-resolved peaks (e.g. Fig. 9A, wild type, 120 min after drug addition) indicate that most cells completed chromosome replication as expected.

**Figure 10.**
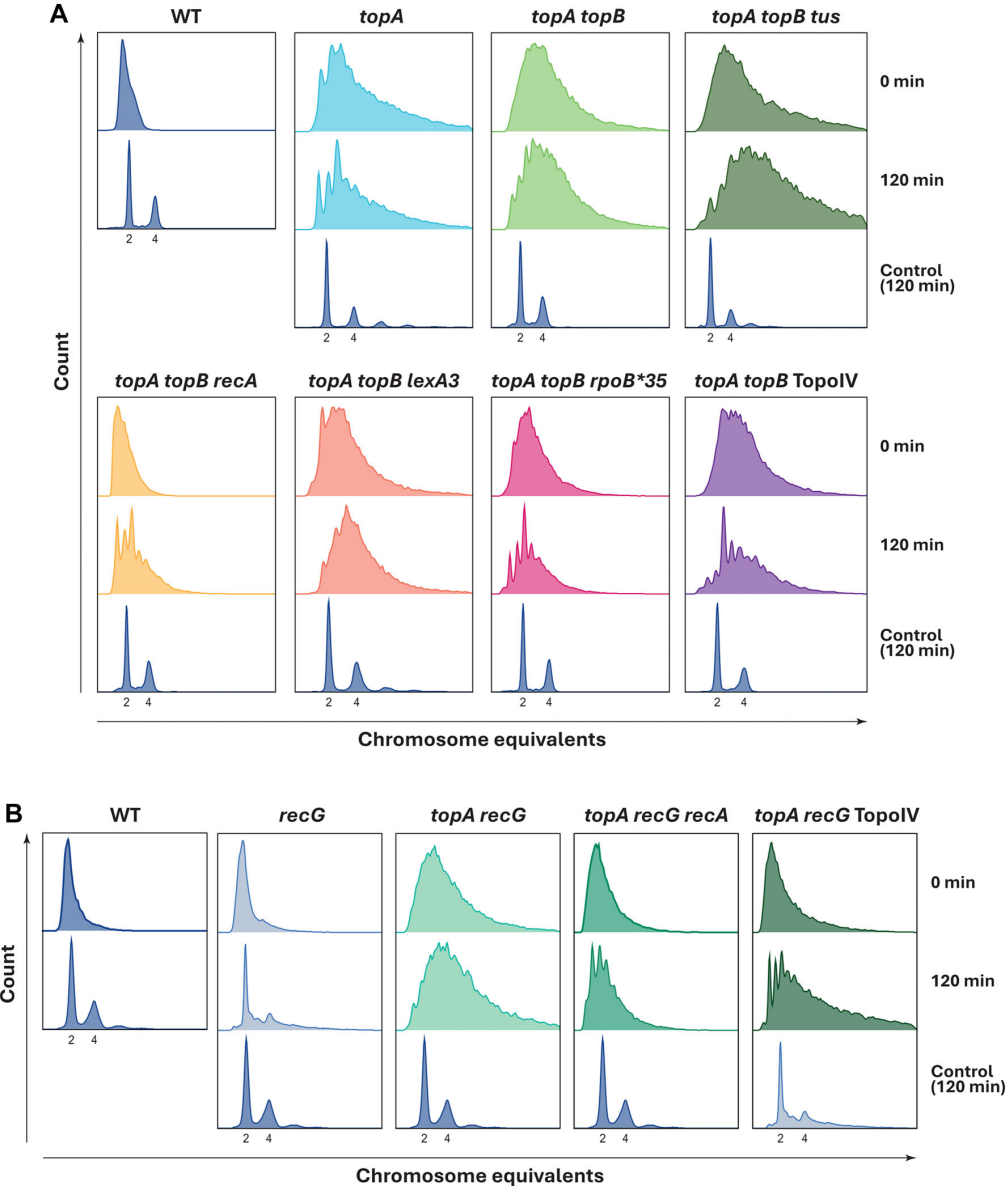
Rifampicin run-out experiments coupled with flow cytometry reveal a severe replication completion defect in *topA topB* and *topA recG* cells. Rifampin run-out experiments for flow cytometry analyses were performed as described in the ‘Materials and methods’ section with cells grown at 30°C. (**A**) RFM443 (WT), JB206 (*topA20::*Tn*10 gyrBts*), JB395 (*topA20::*Tn*10 ΔtopB gyrBts*), JB260 (*topA20::*Tn*10 ΔtopB Δtus gyrBts*), VU243 (*ΔtopA ΔtopB ΔrecA306 srlR301::Tn10 gyrBts*), AD278 (*ΔtopA ΔtopB lexA3 gyrBts*), JB639 (ΔtopA::cam ΔtopB rpoB*35 gyrBts), and JB208 (*topA20::*Tn*10 ΔtopB gyrBts*/pET11-*parCE*) strains. (**B**) RFM443 (WT), VU170 (*ΔrecG*), AD358 (*topA20::*Tn*10 ΔrecG gyrBts*; −topo IV genes amplification), AD458 (*topA20::*Tn*10 ΔrecG ΔrecA1921::spc gyrBts*), and AD360 (*topA20::*Tn*10 ΔrecG recA gyrBts*; + topo IV genes amplification). For each experiment, profiles at 0 and 120 min are shown together with a 120-min control performed in the same experiment. The control was WT strain, except for AD360, for which VU170 (*recG*) strain was used. Numbers 2 and 4 indicate chromosome equivalents. All time points for each experiment are presented in Supplementary Fig. S7.

Figure 10A shows that the *topA* null strain (JB206) displays distinct replication-completed peaks superimposed on a broad base, indicating that a subset of cells failed to properly complete replication. We previously reported that a similar *topA* null mutant exhibits asynchronous replication initiation, as shown by the presence of non-2*n* peaks in minimal medium, consistent with defective regulation of *oriC* [22, 46]. This phenotype likely accounts, at least in part, for the presence of such non-2*n* peaks observed here.

Deletion of *topB* in the *topA* background nearly abolished the discrete peaks, indicating a severe defect in replication completion (Fig. 10A; *topA topB* null strain JB395, 120 min; see Supplementary Fig. S7 for additional time points corresponding to the results shown in Fig. 10). Deletion of *tus* did not restore replication completion in *topA topB* null cells (*topA topB tus* strain JB260), indicating that *Ter*/Tus barriers are not responsible for this defect. In contrast, deletion of *recA* partially alleviated the replication-completion defect (*topA topB recA* strain VU243), whereas preventing SOS induction with the *lexA3* allele did not (*topA topB lexA3* strain AD279). These results indicate that the recombinase activity of RecA, rather than its role in SOS induction, contributes to the replication-completion defect of *topA topB* null cells. As shown in Fig. 6, RecA-dependent replication represents the only detectable replication activity following rifampicin treatment. Furthermore, although a substantial fraction of DSE formation in *topA topB* null cells is Tus-dependent, an even larger fraction is RecA-dependent (Fig. 4). Together, these observations suggest that replication fork collapse followed by RecA-dependent re-replication may occur independently of *Ter*/Tus barriers in *topA topB* null cells, as reported for *recG* mutants [37], and contributes to their replication-completion defect.

Reducing RNAP backtracking has been shown to alleviate transcription–replication conflicts that can otherwise generate DSEs [63, 64]. The widely used rpoB*35 mutation decreases RNAP backtracking and transcription–replication conflicts and has been reported to improve growth and partially correct several phenotypes of *topA* and *topA topB* null strains [32, 45], suggesting that RNAP backtracking is frequent and deleterious in these genetic backgrounds. Moreover, the presence of the rpoB*35 mutation abolishes the positive effect of RNase HI overproduction on the growth of *topA topB* null cells [45]. Here, we show that the rpoB*35 mutation partially alleviates the replication-completion defect of *topA topB* null cells (Fig. 10A, topA topB rpoB*35 strain JB639).

We previously showed that further increasing cellular topo IV levels (from pET11-*parCE*) improves growth and reduces filamentation in *topA topB* null mutants [31], which already moderately overproduce topo IV due to amplification of the *parC–parE* locus. Here, we show that increasing topo IV levels partially restored replication completion in *topA topB* null cells (Fig. 10A, JB208, *topA topB* null cells carrying pET11-*parCE*).

Figure 10B shows a similar replication-completion defect in *topA recG* cells (strain AD358), which is likewise partially suppressed by deletion of *recA* (strain AD458) or by overproduction of topo IV (strain AD360). These results further support the possibility that RecG and topo III cooperate in *E. coli*. Supplementary Fig. S8 shows that deletion of *recQ* in a *topA* background (*topA recQ* strain JB553) neither exacerbated nor alleviated the replication-completion defect observed in *topA* null cells (JB206), once again indicating that RecQ does not function with topo III to correct this problem in *E. coli*.

## Discussion

A previous study attributed the high level of Ter DNA amplification in *topA topB* null mutants to *Ter*/Tus barriers, as in *recG* mutants, rather than to an R-loop-dependent origin of replication in the Ter region [45]. However, the underlying mechanism was not defined, particularly with respect to *Ter*/Tus-dependent DSE formation and the stages at which RecA and topo III might act, making a direct parallel with *recG* mutants difficult to establish. In this work, consistent with the back-and-forth model [37], the strong increase in *Ter*/Tus-dependent DSE formation observed upon deletion of *topB* in *topA* null cells was accompanied by a comparable increase in Ter DNA amplification (likewise bordered by *terA* and *terB*). In this model, iterative cycles of RecA-dependent misoriented replication—each generating a new DSE—drive progressive amplification of the Ter region. In support of this prediction, deletion of *recA* in *topA topB* null cells markedly reduced the accumulation of *Ter*/Tus-dependent DSEs and completely abolished Ter DNA amplification. Moreover, in agreement with Ter amplification arising from RecA-dependent replication (iSDR initiated from D-loops), such replication was detected in *topA topB* null mutants, as is the case in *recG* mutants [62]. Taken together, these findings indicate that topo III, like RecG, suppresses RecA-dependent Ter DNA amplification and led us to propose that topo III acts in concert with RecG to mediate this effect. This interpretation is supported by the finding that deletion of *topB* failed to increase Ter DNA amplification in a *recG* mutant but significantly enhanced it in a *rnhA* mutant, where Ter amplification is initiated by R-loop-dependent replication [31, 34, 35, 60]. Major phenotypes of *topA topB* mutants were also observed in *topA recG* cells: high-level amplification of the *parC* and *parE* genes, formation of highly filamentous cells with diffuse and poorly segregated DNA, and a total failure to complete replication in rifampicin run-out experiments—the latter partially rescued by deleting *recA* or overproducing topo IV.

Single *topB* mutants behave like wild-type cells, exhibiting neither Ter DNA amplification (Supplementary Fig. S4) nor *oriC*-independent replication initiation (SDR) [51]. The absence of Ter DNA amplification in *topB* mutants likely reflects the lack of an alternative mode of replication initiation, in contrast to that observed in *recG, topA*, and *rnhA* mutants. As noted in the Introduction, fragile-site DNA breaks on the *E. coli* chromosome cluster in the Ter region and arise through two distinct mechanisms, one of which involves replication fork collapse at *Ter*/Tus barriers [24]. Fork collapse occurs when a replication fork remains arrested at a *Ter*/Tus barrier long enough for a co-directional fork approaching from behind to collide with it, generating a DSE. Because this process can be detected even in wild-type cells [24]—where replication initiates exclusively at *oriC* and is tightly regulated—it is expected to occur more frequently when replication is also initiated by alternative, cell cycle–independent mechanisms.

Such non-*oriC* replication events include replication initiated following fork fusion in the Ter region, as observed in *recG* mutants [42, 43], as well as replication initiated from R-loops in *rnhA* and *topA* null mutants, some of which may occur within the Ter region [31, 34, 35, 52, 60]. We also note that initiation from both D-loops (iSDR) and R-loops (cSDR) can occur in *recG* mutants [62]. In addition, misregulation of *oriC*-initiated replication in *topA* null mutants [22, 46] may further increase the probability of fork collapse at *Ter*/Tus barriers. Under these conditions, when the putative RecG–topo III pathway is compromised, high-level Ter DNA amplification can occur via the back-and-forth re-replication model, as observed in *recG* single mutants and in *topA topB* and *rnhA topB* double mutants.

How topo III could assist RecG in preventing the re-replication that drives Ter DNA amplification remains difficult to define, largely because the mechanism underlying re-replication itself is still incompletely understood. What is increasingly clear, however, is that re-replication is initiated by RecA-dependent D-loop formation. Both RecG and topo III are capable of acting on D-loop structures. *In vitro*, RecG binds to and unwinds D-loops [65]. Moreover, both *in vitro* and *in vivo*, RecG functions together with PriA at D-loops: RecG promotes the correct mode of PriA binding, which in turn limits replication fork reversal or D-loop destabilization by RecG [37, 66, 67]. A recent systematic analysis of DSE repair following a chromosomal nick in *B. subtilis* established that RecG acts downstream of RecA and immediately upstream of PriA loading, supporting a role for RecG in D-loop remodeling in the DSE repair pathway [68]. In eukaryotes, Rad51—the functional ortholog of RecA—forms D-loops that can be dissociated by the decatenation activity of topo III, with or without the involvement of BLM in human cells or Sgs1 in yeast [69–71]. In *E. coli*, an R-loop formed on a supercoiled plasmid has been identified as a hotspot for topo III activity *in vitro* [72].

**Figure 11.**
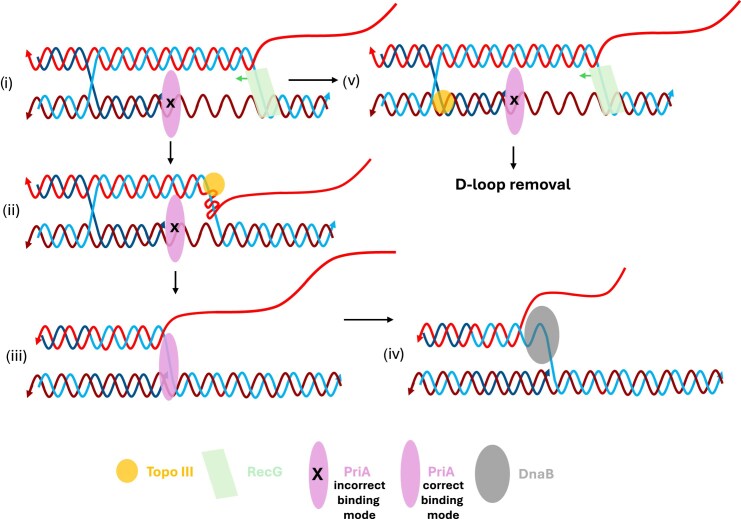
Model for the roles of topo III and RecG in preventing reverse-replication restart. In this model, the D-loop has an extended 5′-ended DNA strand (i). In this configuration, PriA is not bound in the correct mode required for proper DnaB loading (i and [28]) and may therefore be unstable. RecG must remodel the D-loop (ii) to reposition the junction closer to the 3′ end, allowing PriA to switch to its correct, stable, binding mode (iii). However, RecG-mediated remodeling promotes wrapping of the extended 5′-ended strand around the lagging-strand template, which ultimately inhibits further RecG activity. Resolution of this wrapped 5′-ended DNA by topo III (ii) permits RecG to complete D-loop remodeling, enabling correct PriA binding (iii) and subsequent DnaB loading (iv) for replication restart in the proper orientation. Alternatively (v), the decatenation activity of topo III may directly disrupt the D-loop, either independently or in conjunction with RecG. The diagram depicts topo III acting at the D-loop crossing point; alternatively, topo III may efficiently compete with PriA for binding to the invading 3′ end and disrupt the D-loop at this site. See the ‘Discussion’ for more details.

Within the framework of the model proposed by Azeroglu *et al*. [37], processing of a DSE by RecBCD generates an intermediate in which the 5′-ended strand is longer than the 3′-ended one. Consequently, the D-loop must be remodeled by RecG to allow PriA to bind in the correct mode at the junction (Fig. 1B). Figure 11 (i and ii) illustrates a DNA-wrapping problem that depends on the lengths of both the free 5′-ended ssDNA and its annealed region with the lagging-strand template, which may inhibit or substantially slow D-loop remodeling by RecG. Indeed, as RecG unwinds these strands while re-annealing the parental duplex, the long 5′-ended ssDNA can wrap around the lagging-strand template (ii), generating a topological constraint. This constraint can be efficiently resolved by the strong decatenation activity of topo III (ii and iii). Failure to resolve it may hinder RecG-mediated unwinding or subsequent steps, including PriA binding in the correct mode and/or DnaB loading onto the lagging-strand template (ii–iv). A similar untangling reaction has been described in which human topo IIIβ disentangles RNA wrapped around the displaced DNA strand of an R-loop during helicase-mediated R-loop unwinding [73]. Figure 11 (i tov) also illustrates an alternative possibility: because the D-loop cannot be immediately stabilized by PriA, topo III, with or without assistance from RecG helicase activity, may have sufficient time to eliminate it. In this model, topo III would preferentially target D-loops that are permissive for reverse-replication restart.

While this manuscript was being prepared, a study was published demonstrating reverse-replication restart (re-replication) at sites of replication fork collapse caused by ultraviolet-induced pyrimidine dimers in wild-type cells [74]. However, unlike the re-replication observed in *recG* and *topA topB* mutants, this re-replication could be readily detected only when replication from *oriC*—which otherwise masked it—was inhibited. The authors proposed a fork-triplication model in which repair of a DSE by the RecBCD pathway leads to the formation of a D-loop that is permissive for reverse-replication restart [74]. Thus, regardless of the exact mechanism responsible for replication fork reversal, this work further strengthens the conclusion that reverse-replication restart initiates from D-loops.

The inability to complete chromosome replication observed in *topA topB* and *topA recG* mutants is already present in the *topA* null mutant and is exacerbated by deletion of either *topB* or *recG*. This phenotype is partially suppressed in the *topA topB* null mutant by the *rpoB*35 mutation, which makes the RNAP backtracking-resistant. This mutation has been shown to improve the growth of *topA* and *topA topB* null mutants and to markedly reduce R-loop-dependent replication [32, 45]. Moreover, *rpoB*35 also suppresses several phenotypes of *recG* mutants [35, 63]. We propose that *topA* null mutants experience severe transcription–replication conflicts driven by excessive RNAP backtracking and associated R-loop formation [45], which constitute stable barriers to replication fork progression. These obstacles strongly impair completion of chromosome replication and can lead to the formation of DSEs. When *topB* or *recG* is deleted in a *topA* background, many of these DSEs are improperly repaired, resulting in replication restart in the wrong orientation (re-replication). Both re-replication itself and the ensuing transcription–replication conflicts further aggravate the replication-completion defect. Partial suppression of this phenotype by deletion of *recA* is consistent with this model. However, suppression by the *rpoB*35 mutation is only partial, indicating that additional sources of DNA damage contribute to the observed phenotypes.

RecG and RecQ, as well as eukaryotic homologs of *E. coli* RecQ such as BLM and Sgs1, belong to helicase superfamily 2 (SF2), the largest and most diverse helicase group characterized by conserved ATP-dependent motor domains that remodel nucleic acid structures [75]. As noted in the Introduction, unlike BLM and Sgs1, which act with Topo III during recombination, evidence for RecQ acting with Topo III in recombination in *E. coli* has been lacking. In fact, our results indicate that RecQ does not act with Topo III and instead identify RecG as the helicase acting with Topo III during replication-associated hyper-recombination. This is consistent with biochemical studies showing that BLM, like RecG but unlike RecQ, efficiently remodels branched and multistranded DNA structures, including replication fork–like substrates [27, 76–79]. Both proteins also efficiently bind to and promote branch migration of HJs [80–83]. Moreover, expression of *E. coli recG* partially suppresses the phenotype of BLM-deficient human cell lines [23].

In conclusion, this work suggests a role for topo III in acting with RecG, but not RecQ, on RecA-generated D-loops during DSE repair to prevent Ter DNA amplification and to promote completion of chromosome replication. Both RecG and topo III localize to replication forks, at least in part through their established interactions with the SSB (single-stranded DNA-binding) protein [19, 84, 85]. These interactions with SSB are likely to facilitate their activities in suppressing re-replication from D-loops. Further studies will be required to define the functional relationship between RecG and topo III and to clarify its broader role in DNA metabolism.

## Supplementary Material

gkag572_Supplemental_File

## Data Availability

Sequencing data from Illumina whole genome sequencing are available in the Sequence Read Archive under BioProject accession number PRJNA1432067.
