# Supplementary material for: Topoisomerase III limits RecA-dependent DNA amplification in the chromosome terminus with RecG
Source: Nucleic Acids Res. 2026 Jun 8;54(11):gkag572. doi: 10.1093/nar/gkag572 (PMC13244152; doi:10.1093/nar/gkag572)
Supplement: gkag572_Supplemental_File [file gkag572_supplemental_file.pdf]

Supplementary Material

Topoisomerase III limits RecA-dependent DNA amplification in the chromosome terminus with RecG.

Ali Dadras[1], Armelle Le Campion[2] and Marc Drolet[1,2,*]

[1]Département de microbiologie, infectiologie et immunologie, Faculté de médecine, Université de Montréal, Montréal, P. Québec, Canada, H3C 3J7.

[2]Institut Courtois d'innovation biomédicale, Faculté de médecine, Université de Montréal, Montréal, P. Québec, Canada, H3C 3J7.

*Corresponding author
Email: marc.drolet@umontreal.ca

Supplementary table

Table S1. *Escherichia coli K12* strains and plasmids used in this work.

The strains were constructed as described in Materials and Methods.

Strain or plasmid Name	*Genotype or Relevant Genotype*	*Reference or Source*
AD20	JB393 P_{N25}*tetR* FRTKanFRT	JB393 × P1 SMR14323
AD45	JB393 P_{N25}*tetR* FRTKanFRT::FRT*cat*FRT P_{N25}*tetO gam-gfp*	AD20 × P1 SMR14334
AD50	RFM445 P_{N25}*tetR* FRTKanFRT	RFM445 × P1 SMR14323
AD58	RFM445 P_{N25}*tetR* FRTKanFRT::FRT*cat*FRT P_{N25}*tetO gam-gfp*	AD50 × P1 SMR14334
AD80	RFM445 *ΔtopB ΔtusB topA20::*Tn*10* P_{N25}*tetR* FRTKanFRT	JB260 × P1 SMR14323
AD85	RFM445 *ΔtopB ΔtusB topA20::*Tn*10* P_{N25}*tetR* FRTKanFRT::FRT*cat*FRT P_{N25}*tetO gam-gfp*	AD80 × P1 SMR14334
AD88	JB395 P_{N25}*tetR* FRTKanFRT	JB395 × P1 SMR14323
AD95	JB395 P_{N25}*tetR* FRTKanFRT::FRT*cat*FRT P_{N25}*tetO gam-gfp*	AD88 × P1 SMR14334
AD100	JB395 *ΔrecO737::kan*	JB395 × P1 VU462
AD115	RFM443 pEAW915	This work
AD130	JB395 pEAW915	This work
AD145	RFM445 pEAW915	This work
AD165	JB260 pEAW915	This work
AD278	RFM475 *ΔtopB lexA3 malF::Tn10*	VU217 × P1 VU500
AD279	RFM475 *ΔtopB lexA3 malF::Tn10*	VU217 × P1 VU500
AD294	AD95 *ΔrecA1921::spc*	AD95 × P1 AM1992
AD320	JB206 pEAW915	This work
AD327	MM69 *kan* removed	MM69, *kan* removed by pCP20
AD330	AD327 *Δtus758::kan*	AD327 × P1 VU490
AD357[1]	JB206 *ΔrecG::kan*	JB206 × P1 VU170
AD358[1]	JB206 *ΔrecG::kan*	JB206 × P1 VU170
AD359[1]	JB206 *ΔrecG::kan*	JB206 × P1 VU170
AD360[1]	JB206 *ΔrecG::kan*	JB206 × P1 VU170
AD361[1]	JB206 *ΔrecG::kan*	JB206 × P1 VU170
AD362[1]	JB206 *ΔrecG::kan*	JB206 × P1 VU170
AD373	RFM445 *ΔrnhA::cam*	RFM445 × P1 MM84
AD378[2]	VU409 *ΔrnhA::cam*	VU409 × P1 MM84
AD382[2]	VU409 *ΔrnhA::cam*	VU409 × P1 MM84

AD398[3]	VU409 *ΔrecG::kan*	VU409 × P1 VU170
AD400[3]	VU409 *ΔrecG::kan*	VU409 × P1 VU170
AD404[4]	RFM445 *ΔrecG::kan*	RFM445 × P1 VU170
AD405[4]	RFM445 *ΔrecG::kan*	RFM445 × P1 VU170
AD458	AD358 *ΔrecA1921::spc*	AD358 × P1 AM1992
AM1992	F-, λ^-, *ΔrecA1921::spc*, *rph-1*	(1)
CT47	*ΔthyA36, deoC2, IN(rrnD-rrnE)I, rph?, recQ6215 (sub cat 883 for cdn 19-606), recQ6215::cam*	Lab collection
CT170	RFM475 *ΔtopB*	(2)
JB206	RFM445 *topA20::*Tn*10*	(3)
JB208	RFM445 *ΔtopB topA20::*Tn*10* pET11-*parEC*	(3)
JB260	RFM445 *ΔtopB ΔtusB topA20::*Tn*10*	(3)
JB393	RFM445 *ΔtopB topA20::*Tn*10* pSK760	(3)
JB395	RFM445 *ΔtopB topA20::*Tn*10* pSK762c	(3)
JB535	RFM445 *recQ6215::cam*	RFM445 x P1 CT47
JB553	JB535 *topA20::*Tn*10*	JB535 × P1 JB206
JB639	RFM445 *rpo*35 btuB*::Tn*10 ΔtopB ΔtopA::cam*	(3)
MM68	RFM443 P_{N25}*tetR* FRTKanFRT::FRT*cat*FRT P_{N25}*tetO gam-gfp*	RFM443 × P1 SMR14323 × P1 SMR14334
MM69	RFM475 P_{N25}*tetR* FRTKanFRT::FRT*cat*FRT P_{N25}*tetO gam-gfp*	RFM475 × P1 SMR14323 × P1 SMR14334
MM84	RFM443 *ΔrnhA::cam*	(4)
RFM443	*Δ(codB-lacI)3 rpsL200 galK2*(Oc) *IN(rrnD-rrnE)1 rph-1*	(5)
RFM445	*Δ(codB-lacI)3 rpsL200 galK2*(Oc) *IN(rrnD-rrnE)1 rph-1 gyrB221* (Cou^r) *gyrB203*(Ts)	(5)
RFM475	*rpsL galK2 gyrB221* (Cou^r) *gyrB203*(Ts) *Δlac74 Δ(topA cysB)204*	(5)
SMR14323	MG1655 *ΔaraBAD567 Δattλ*::P_{BAD} *zfd2509.2*::P_{N25}*tetR* FRTKanFRT	(6)
SMR14334	MG1655 *ΔaraBAD567 Δattλ*::P_{BAD} *zfd2509.2*::P_{N25}*tetR* FRT *ΔattTn7*::FRT*cat*FRT P_{N25}*tetO gam-gfp*	(6)
VU170	JW3627-110665 (*ΔrecG::kan*)	(7)
VU205	RFM475 *recQ6215::cam ΔtopB::kan*	(8)
VU217	CT170 (RFM475 *ΔtopB*)	(8)
VU243	RFM475 *ΔtopB ΔrecA306 srlR301::Tn10*	(8)
VU409	RFM445 *ΔtopB*	(8)
VU500	MG1655 *lexA3::malFTn10*	Lab collection
VU462	JW2549-1 (*ΔrecO737::kan*)	(7)
VU490	JW1602-3 (*Δtus758::kan*)	(7)
pCP20	FLP helper plasmid for removal of Km^R cassette	(9)
pEAW915	P*recN-gfp* (Cm^R) for use as an SOS reporter	(10)
pSK760	*rnhA* gene with its own promoter	(5)
pSK762c	like pSK760 but *rnhA* is mutated and inactive	(5)

1- Independent *ΔrecG::kan* transductants (clones) of JB206, obtained in the same experiment.
2- Independent *ΔrnhA::cam* transductants (clones) of VU409, obtained in the same experiment.
3- Independent *ΔrecG::kan* transductants (clones) of VU409, obtained in the same experiment.
4- Independent *ΔrecG::kan* transductants (clones) of RFM445, obtained in the same experiment.

Supplementary Figures

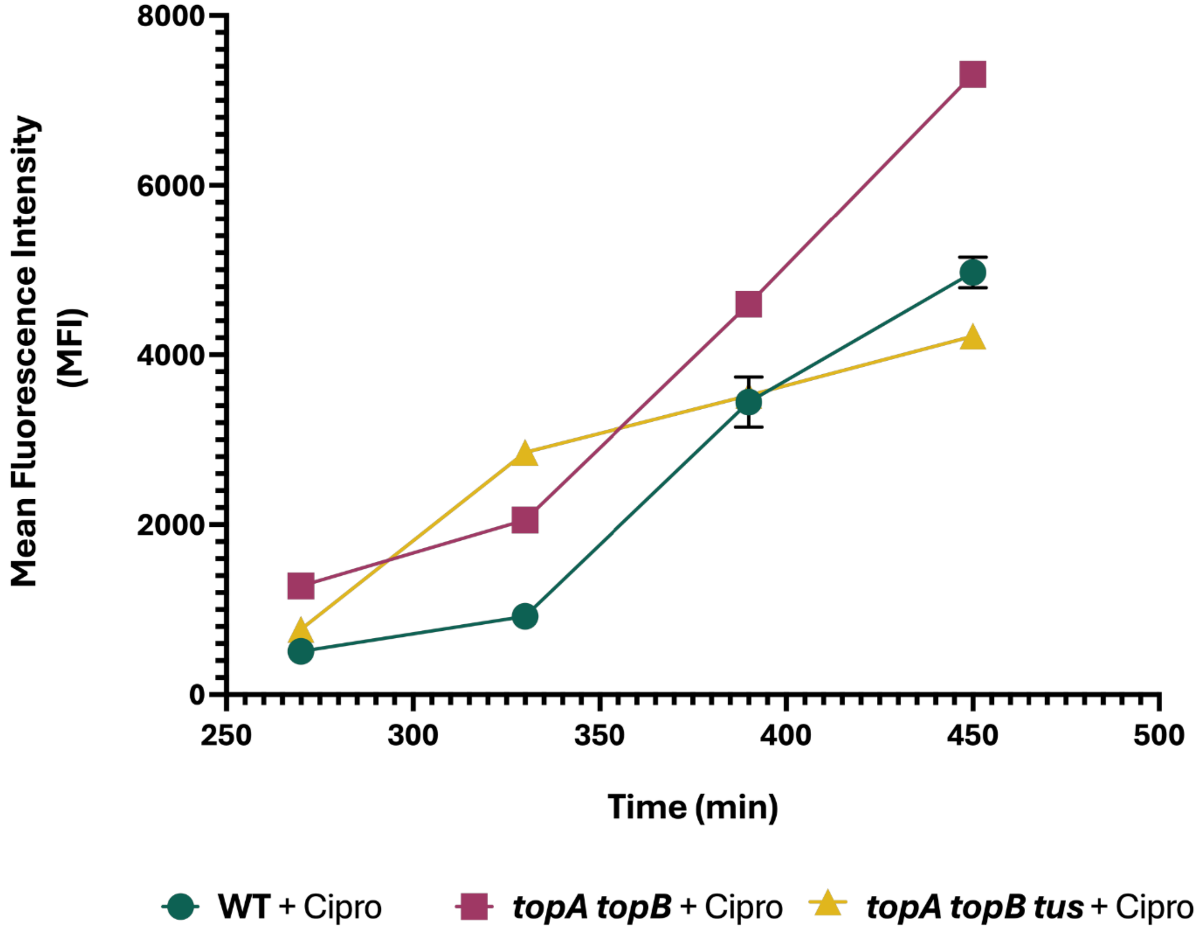

Figure S1. Detection of the ciprofloxacin-induced SOS response using the SOS reporter plasmid pEAW915. RFM443 (WT), JB395 (*topA topB gyrBts*), and JB260 (*topA topB tus gyrBts*) cells carrying the SOS reporter plasmid pEAW915 were grown at 30 °C and prepared for flow cytometry as described in Materials and Methods. Ciprofloxacin (50 ng/ml) was added at 270 min (the first time point shown). The graph shows the average level of SOS induction per cell over time, expressed as mean fluorescence intensity.

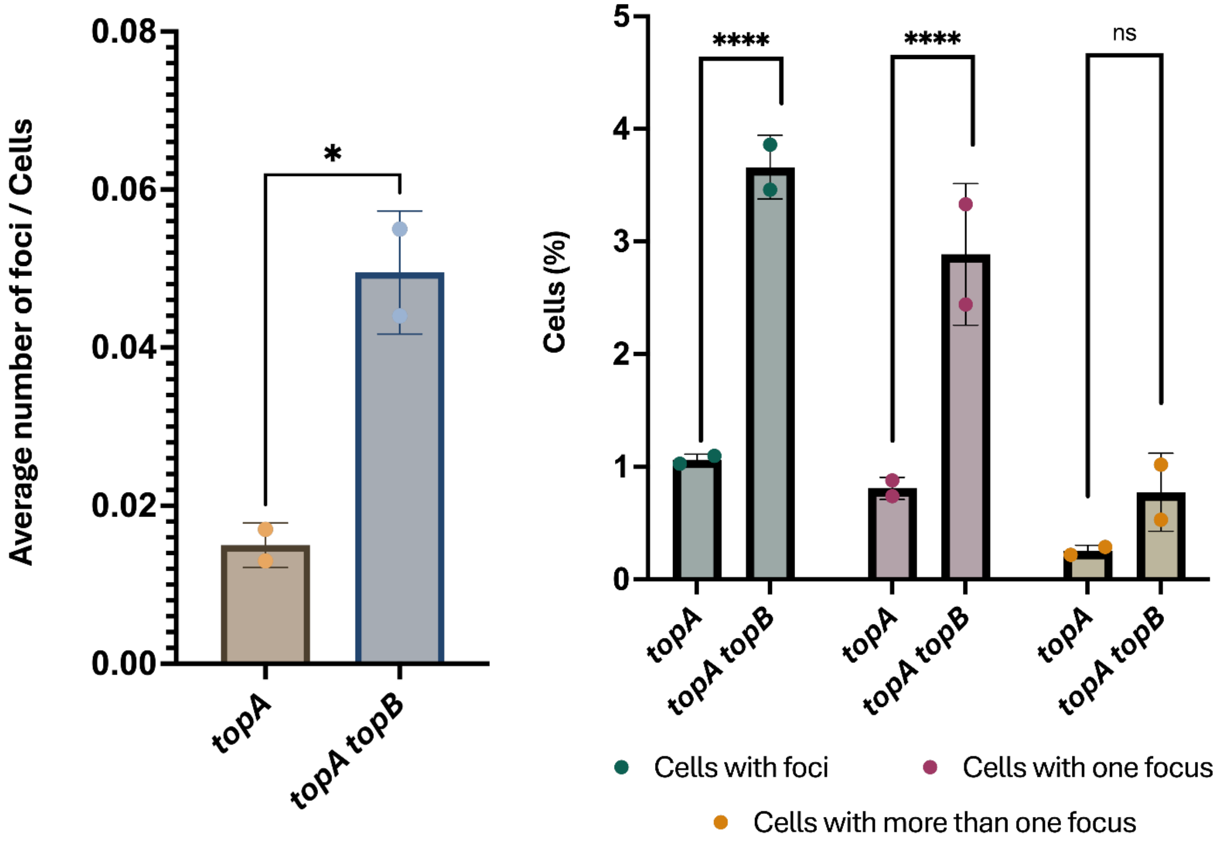

Figure S2. Gam-GFP reporter fusion to detect DSEs in *topA* and *topA topB* strains grown at 37ºC. MM69 (*topA gyrBts*) and AD95 (*topA topB gyrBts*) strains carrying a Gam–GFP reporter fusion were grown at 37°C and prepared for fluorescence microscopy to visualize and quantify Gam–GFP foci, as described in Materials and Methods. The total number of cells analyzed and the number of independent experiments for each strain were as follows: *topA*, 3,566 cells (n = 2) and *topA topB*, 1,553 cells (n = 2). The left histogram shows the mean number of foci per cell, while the right histogram shows the percentage of cells containing foci, including cells with a single focus and those with multiple foci.

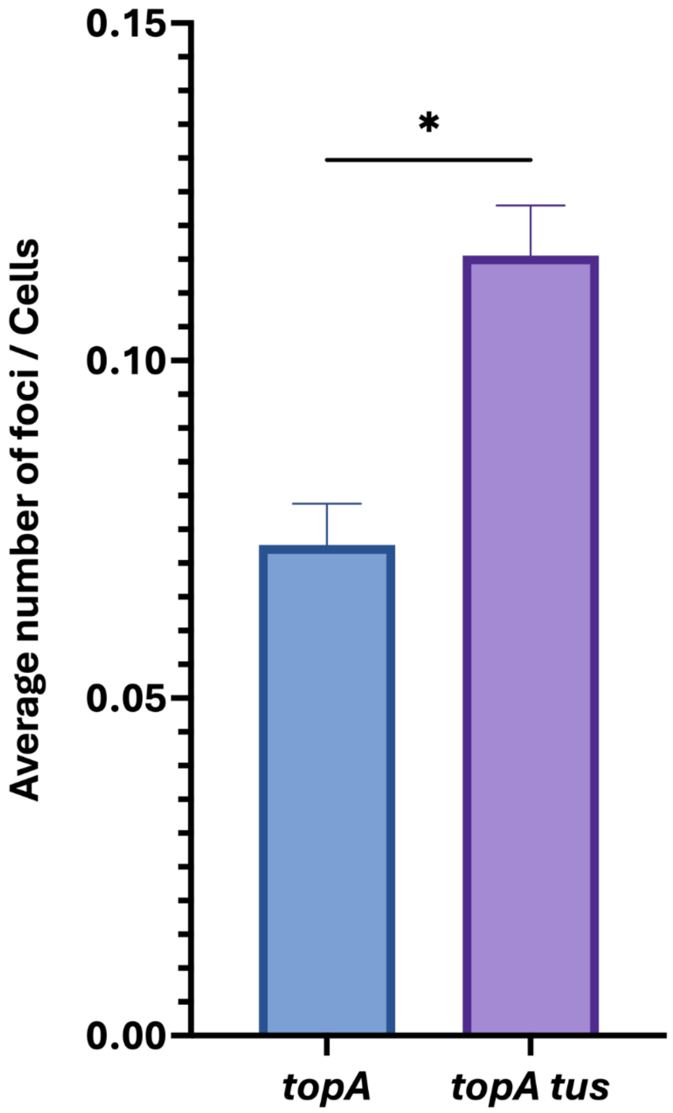

Figure S3. Gam-GFP reporter fusion to detect DSEs in strains *topA* and *topA tus* strains. MM69 (*topA gyrBts*) and AD330 (*topA tus gyrBts*) strains carrying a Gam–GFP reporter fusion were grown at 30°C and prepared for fluorescence microscopy to visualize and quantify Gam–GFP foci, as described in Materials and Methods. The total number of cells analyzed and the number of independent experiments for each strain are: *topA*, 5,273 cells (n = 2) and *topA tus*, 4,157 cells (n = 2). The histogram shows the mean number of foci per cell.

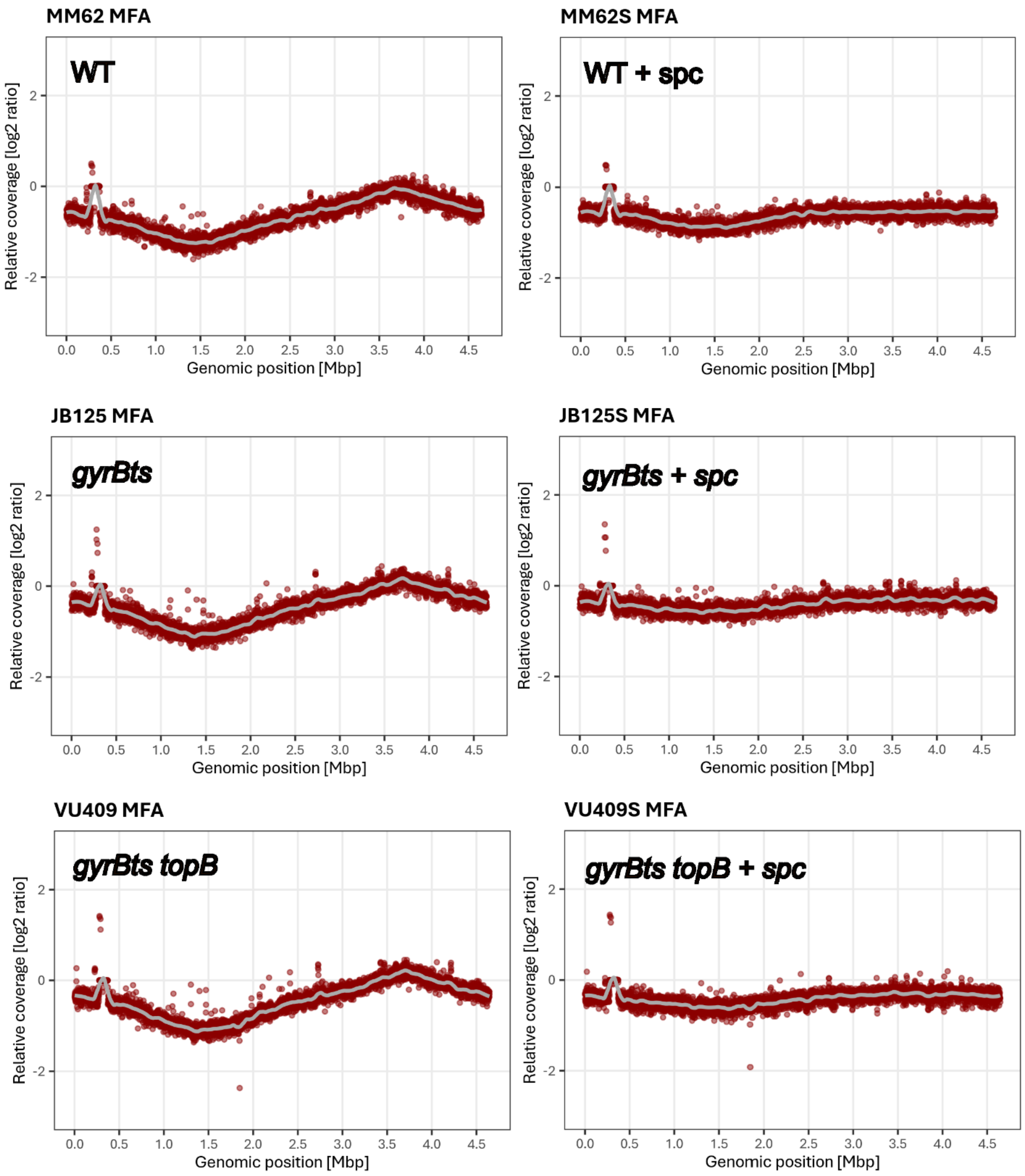

Figure S4. MFA replication profiles of control strains. RFM443 (WT), RFM445 (*gyrBts*), and VU409 (*topB gyrBts*) cells were grown at 30 °C to logarithmic phase and treated or not with spectinomycin (+spc) for 2 h prior to genomic DNA extraction, as described in Materials and Methods. See the legend to Fig. 2 for additional details. *oriC* and *ter* indicate the normal origin of replication and the site of replication termination (fork convergence), respectively. The strain name is indicated above each MFA diagram and is followed by the letter S, denoting spectinomycin addition during growth.

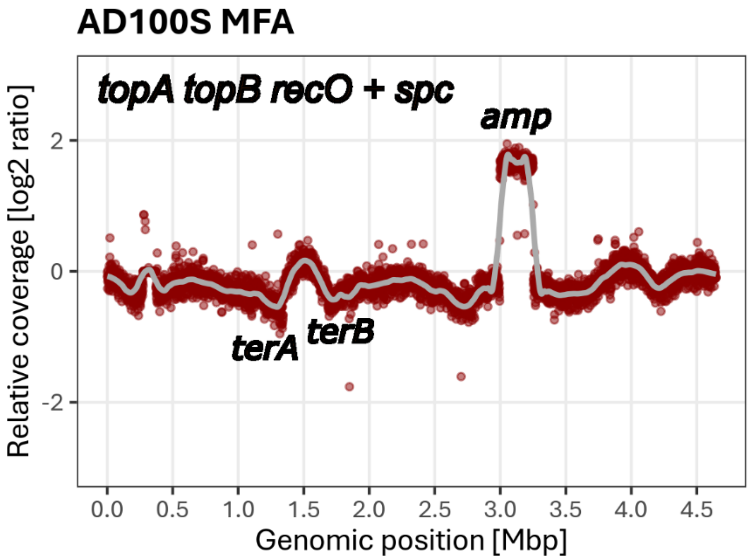

Figure S5. MFA replication profile of a *topA topB recO* strain. AD100 (*topB recO gyrBts*) cells were grown at 30 °C to logarithmic phase and treated with spectinomycin for 2 h prior to genomic DNA extraction, as described in Materials and Methods. See the legend to Fig. 2 for additional details. The positions of the *TerA* and *TerB* barriers as well as the amplified *parC* and *parE* region *(amp*) are indicated.

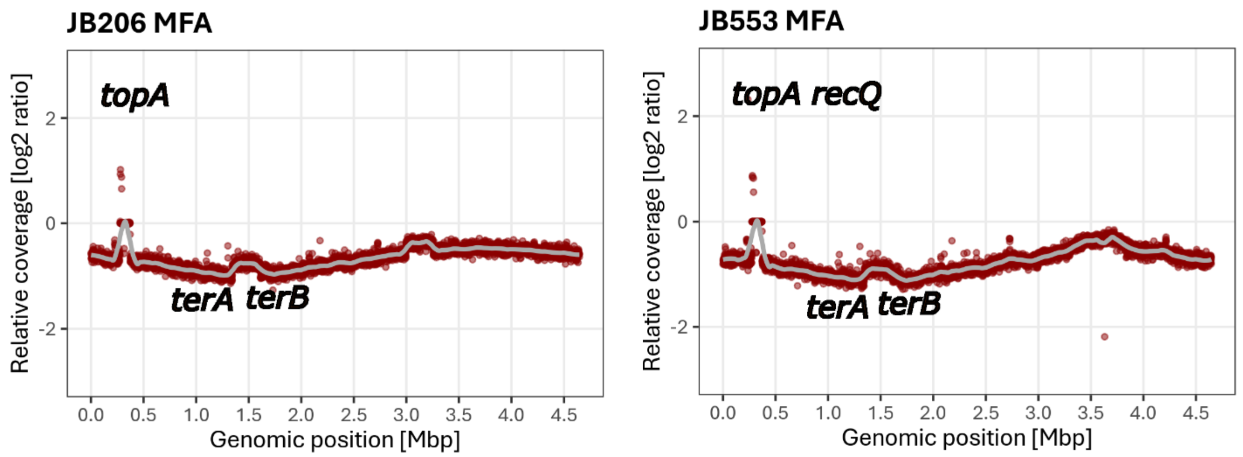

Figure S6. MFA replication profiles of *topA* and *topA recQ* strains. JB206 (*topA gyrBts*) and JB553 (*topA recQ gyrBts*) cells were grown at 30 °C to logarithmic phase without spectinomycin and processed for genomic DNA extraction as described in Materials and Methods. The positions of the *TerA* and *TerB* barriers, as well as the amplified *parC–parE* region (*amp*), are indicated. See the legend to Fig. 2 for additional details.

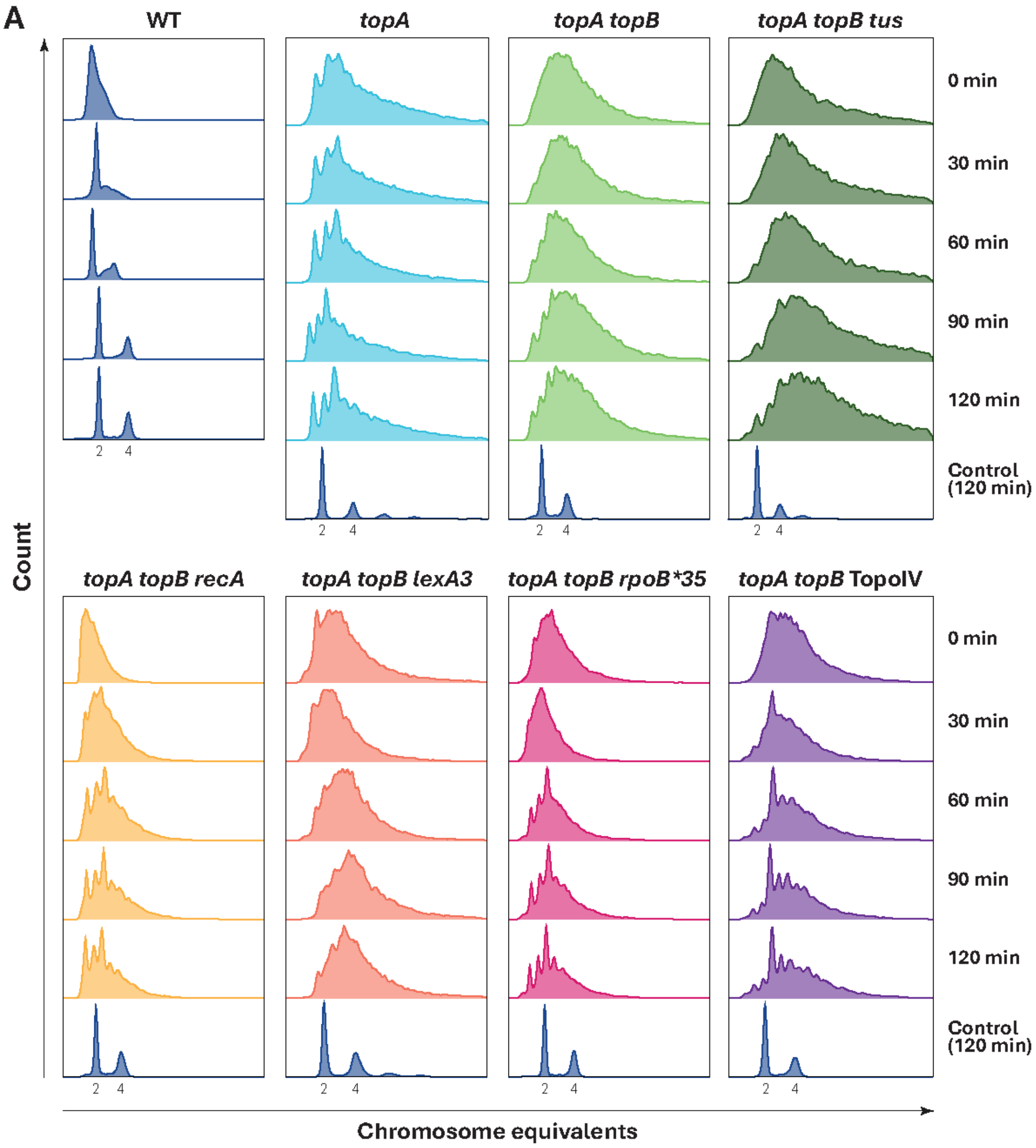
A
WT
topA
topA topB
topA topB tus
0 min
30 min
60 min
90 min
120 min
Control (120 min)
Count
topA topB recA
topA topB lexA3
topA topB rpoB*35
topA topB TopoIV
2
4
Chromosome equivalents

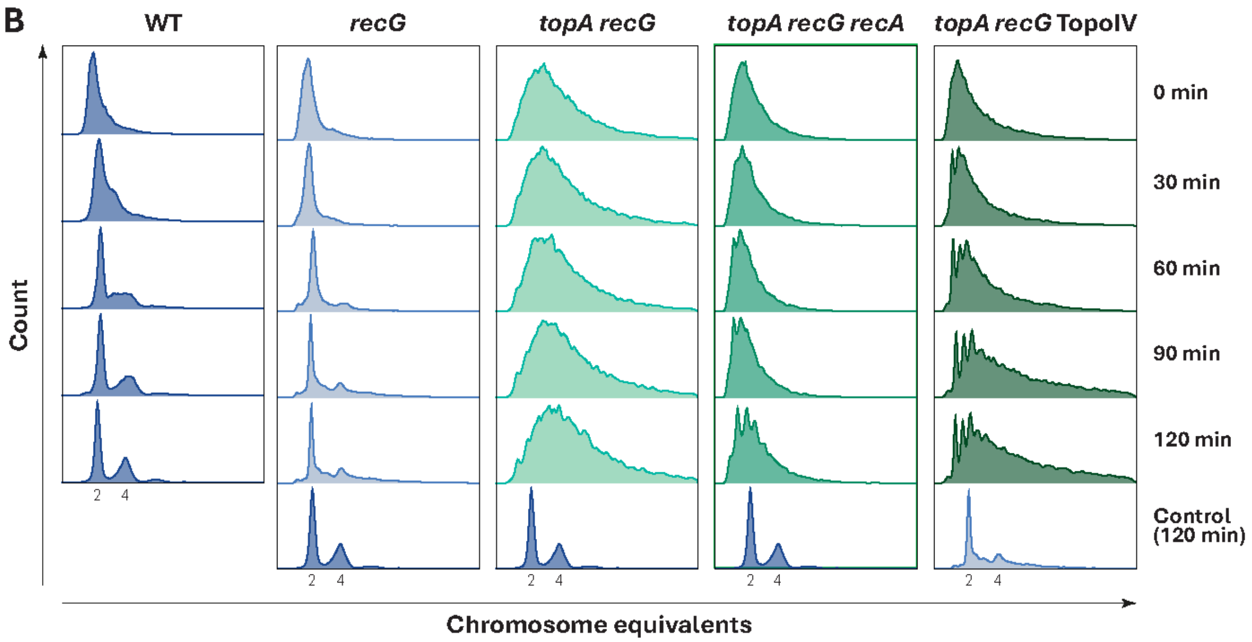

Figure S7. Rifampicin run-out experiments coupled with flow cytometry reveal a severe replication-completion defect in *topA topB* and *topA recG* cells. All time points from the rifampicin run-out experiments shown in Fig. 9A are presented in **(A)**, and all time points from those shown in Fig. 9B are presented in **(B)**. See the legend to Fig. 9 for additional experimental details.

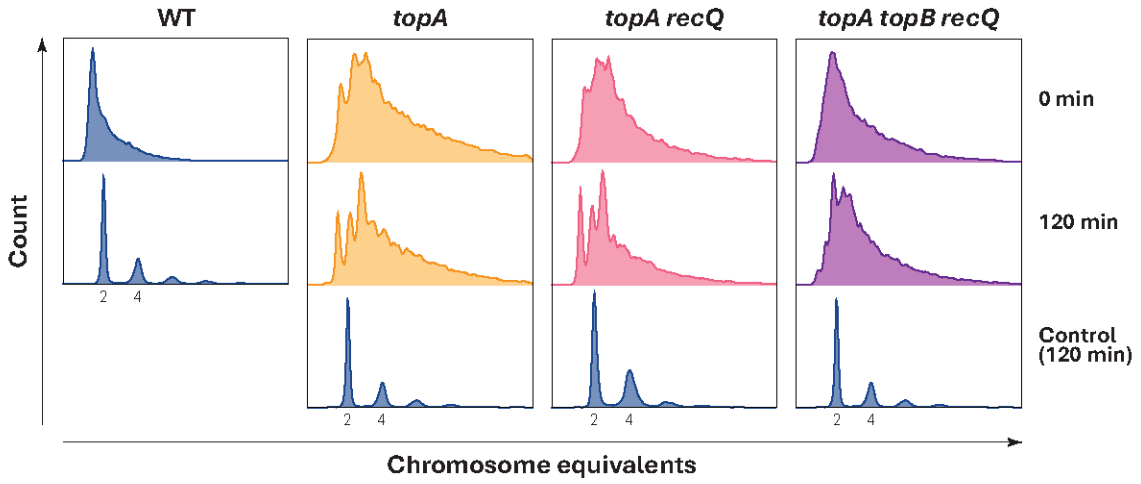

Figure S8. Rifampicin run-out experiments coupled with flow cytometry showing the effect of *recQ* deletion on replication completion in type IA topos mutants. in *topA* and *topA recQ* strains. Rifampicin run-out experiments were performed as described in Materials and Methods. Data are shown for JB206 (*topA gyrBts*; reproduced from Fig. 9), JB553 (*topA recQ gyrBts*), and VU205 (*topA topB recQ gyrBts*). For each experiment, profiles at 0 and 120 min are shown together with a 120-min WT control performed in parallel. Numbers 2 and 4 indicate chromosome equivalents.

Supplementary references

1. Park, K.T., Du, S. and Lutkenhaus, J. (2020) Essential Role for FtsL in Activation of Septal Peptidoglycan Synthesis. *mBio*, **11**.
2. Usongo, V., Tanguay, C., Nolent, F., Bessong, J.E. and Drolet, M. (2013) Interplay between type 1A topoisomerases and gyrase in chromosome segregation in Escherichia coli. *J Bacteriol*, **195**, 1758-1768.
3. Brochu, J., Vlachos-Breton, E., Irsenco, D. and Drolet, M. (2023) Characterization of a pathway of genomic instability induced by R-loops and its regulation by topoisomerases in E. coli. *PLoS Genet*, **19**, e1010754.
4. Brochu, J., Vlachos-Breton, E., Sutherland, S., Martel, M. and Drolet, M. (2018) Topoisomerases I and III inhibit R-loop formation to prevent unregulated replication in the chromosomal Ter region of Escherichia coli. *PLoS Genet*, **14**, e1007668.
5. Drolet, M., Phoenix, P., Menzel, R., Masse, E., Liu, L.F. and Crouch, R.J. (1995) Overexpression of RNase H partially complements the growth defect of an Escherichia coli delta topA mutant: R-loop formation is a major problem in the absence of DNA topoisomerase I. *Proc Natl Acad Sci U S A*, **92**, 3526-3530.
6. Shee, C., Cox, B.D., Gu, F., Luengas, E.M., Joshi, M.C., Chiu, L.Y., Magnan, D., Halliday, J.A., Frisch, R.L., Gibson, J.L. *et al.* (2013) Engineered proteins detect spontaneous DNA breakage in human and bacterial cells. *Elife*, **2**, e01222.
7. Baba, T., Ara, T., Hasegawa, M., Takai, Y., Okumura, Y., Baba, M., Datsenko, K.A., Tomita, M., Wanner, B.L. and Mori, H. (2006) Construction of Escherichia coli K-12 in-frame, single-gene knockout mutants: the Keio collection. *Mol Syst Biol*, **2**, 2006 0008.
8. Usongo, V. and Drolet, M. (2014) Roles of type 1A topoisomerases in genome maintenance in Escherichia coli. *PLoS Genet*, **10**, e1004543.
9. Datsenko, K.A. and Wanner, B.L. (2000) One-step inactivation of chromosomal genes in Escherichia coli K-12 using PCR products. *Proc Natl Acad Sci U S A*, **97**, 6640-6645.
10. Ronayne, E.A., Wan, Y.C., Boudreau, B.A., Landick, R. and Cox, M.M. (2016) P1 Ref Endonuclease: A Molecular Mechanism for Phage-Enhanced Antibiotic Lethality. *PLoS Genet*, **12**, e1005797.
